# Global analysis of the energy landscapes of molecular crystal structures by applying the threshold algorithm

**DOI:** 10.1038/s42004-022-00705-4

**Published:** 2022-07-28

**Authors:** Shiyue Yang, Graeme M. Day

**Affiliations:** School of Chemistry, University of Southampton, Southampton, SO17 1BJ UK

**Keywords:** Structure prediction, Materials chemistry, Computational chemistry, Computational methods, Crystal engineering

## Abstract

Polymorphism in molecular crystals has important consequences for the control of materials properties and our understanding of crystallization. Computational methods, including crystal structure prediction, have provided important insight into polymorphism, but have usually been limited to assessing the relative energies of structures. We describe the implementation of the Monte Carlo threshold algorithm as a method to provide an estimate of the energy barriers separating crystal structures. By sampling the local energy minima accessible from multiple starting structures, the simulations yield a global picture of the crystal energy landscapes and provide valuable information on the depth of the energy minima associated with crystal structures. We present results from applying the threshold algorithm to four polymorphic organic molecular crystals, examine the influence of applying space group symmetry constraints during the simulations, and discuss the relationship between the structure of the energy landscape and the intermolecular interactions present in the crystals.

## Abstract (web summary)

Current crystal structure prediction methods for anticipating polymorphism are lacking in their ability to provide information on the depth of each energy minimum and the possible transition paths and energy barriers between polymorph structures. Here, a Monte Carlo threshold algorithm is applied to four polymorphic organic systems to estimate the energy barriers that separate the different polymorph structures and characterise the global structure of the energy landscapes of their molecular crystal structures.

## Introduction

Computational approaches for exploring the energy landscapes of molecular crystals continue to develop rapidly as applications of crystal structure prediction (CSP) methods expand beyond the main application of anticipating pharmaceutical polymorphs^1–3^ into screening of co-crystals^4^ and solvates^5^, and incorporation of CSP into computer-guided discovery of functional materials^6–10^.

CSP typically relies on an exploration for the local energy minima on the high-dimensional energy surface as a function of the structural variables that determine the packing of a molecule into a crystal^11^. The structures corresponding to each local energy minimum are usually considered as possible polymorphs, with the assumption that the lowest energy predicted structures correspond to the most likely candidates to be observed experimentally. One limitation of the output of such methods is that, while they provide the geometry and energy of each potential structure, no information is gained about the depth of each energy minimum, nor possible transition paths and energy barriers between structures. This is currently a limiting factor in the analysis of the results of CSP.

One reason for needing a more global picture of the crystal energy landscape is to distinguish between structures occupying deep and shallow energy minima, i.e., are the energy barriers surrounding the structure large or small? An important observation that has been made is that structures corresponding to known polymorphs are often connected to multiple, shallow energy minima by small energy barriers^12^; traditional CSP methods would suggest each of these minima as possible alternative polymorphs, while a knowledge of small energy barriers separating such structures would show that they can merge into fewer distinct structures due to thermal energy at the temperature of interest. Thus, not distinguishing between deep and shallow energy minima contributes to the over-prediction of polymorphism^13^.

Another area of particular interest is the identification of crystal structures that do not correspond to the thermodynamically most stable structure, but occupy sufficiently deep energy basins to be isolable and kinetically stable. Knowing about such structures is important for anticipating polymorphism that could occur through crystallization routes where kinetics can lead the crystal structure away from the thermodynamically preferred, global energy minimum. One example of such a process is the desolvation of solvate crystal structures^14,15^, where solvent incorporated into the crystal structure stabilizes an alternative arrangement of molecules, so that removal of solvent leaves the structure in a metastable polymorph. Some recent studies^6,8,16^ have identified very high energy polymorphs through desolvation of solvates. The importance of these structures is demonstrated by their very attractive properties, such as for high gas storage capacity, selectivity for molecular separations^6^ and high photocatalytic activity^8^.

Molecular dynamics approaches have been applied to study the transitions between polymorphs^17^ and, in the context of CSP, to identify structures that interconvert at non-zero temperatures^18–21^ and methods using metadynamics can also quantify the barriers between structures^22,23^. In this study, we present the implementation of the threshold algorithm, which is based on Monte Carlo sampling of the energy landscape^24^, to molecular crystals, using an accurate force field with an atomic multipole description of electrostatics. The aim of the threshold algorithm is to find the lowest energy at which transitions are possible between local energy minima. By combining trajectories from multiple starting structures, a global picture of the connectivity of minima can be constructed. The threshold algorithm has previously been applied to fairly simple inorganic crystal structures, to investigate the energy landscape of MgF_2_^25^ and to study the entropic stabilization of high energy phases of CaF2^26^. Here, we investigate its application to more complex molecular organic crystals, which are characterized by a balance of several types of intermolecular interactions, and discuss the insight that this method provides to help understand their polymorphism.

## Results and discussion

### Choice of systems

**Fig. 1:**
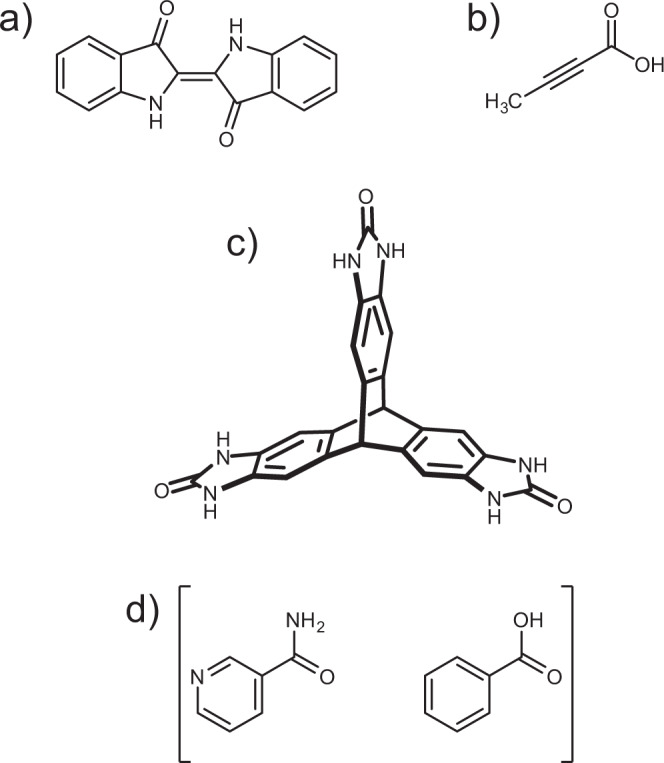
Molecular crystal systems studied in this work. **a** Indigo, **b** tetrolic acid, **c** TTBI and **d** the 1:1 co-crystal of nicotinamide and benzoic acid (GAZCES).

Four crystal systems (Fig. 1), including single-component crystals and a co-crystal, were chosen for study, each of which has known polymorphism. All molecules have reasonably rigid molecular structures, so that rigid-molecule simulations should provide a realistic picture of the crystal energy landscape.

Indigo and tetrolic acid are both small, hydrogen bonding molecules, each with two known polymorphs. Both indigo polymorphs have the same space group symmetry and the same network of hydrogen bonds. In contrast, the polymorphs of tetrolic acid occupy different space groups and differ in hydrogen bonding. We also study the co-crystal formed between nicotinamide and benzoic acid, which is referred to by the Cambridge Structural Database^27^ reference code of its known crystal structure^28^, GAZCES. The co-crystal system has both experimental structures in the same space group, *P*2_1_/*c*, but with changes in the arrangement of hydrogen bonds. Triptycene trisbenzimidazolone (TTBI), has a more complex energy landscape and five experimentally observed polymorphs distributed over a wide energy range.

These differences allow us to test how changes in the network of strong intermolecular interactions are reflected in the energy landscape. The systems with known structures in the same or in different space groups are used to assess sampling with and without symmetry constraints.

### Computational details: the threshold algorithm and disconnectivity graph

The threshold algorithm was developed as a method for finding the energy barrier between structures without the requirement of energy gradient and Hessian matrix calculations^24,29^. Initiated from a local minimum on the energy landscape, a Monte Carlo trial is generated by random local perturbations with the restriction that the single point (i.e. unminimized) energy of the perturbed structure is below a defined threshold energy, called the lid energy. All attempted moves that remain below the current lid energy are accepted and all moves that increase the energy above the lid energy are rejected. Thus, the trajectory can only explore a local pocket on the lattice energy surface and can never reach regions with energy higher than the lid. If the energy barrier between the current structure and another is higher than the lid, the transition between the two energy basins in which these structures are located cannot be sampled.

After a period of simulation, the lid energy is shifted to higher energy to increase the configurational space that is available to the trajectory, allowing transitions to new structures separated by energy barriers lower than the new energy lid. Therefore, when a trajectory visits the energy basin of a new local minimum, the energy barrier between the new and initial minima can be estimated as the current energy lid. An assumption here is that the step size of allowed perturbations is small enough that attempted moves cannot jump through energy barriers. The sampling under each energy lid needs, in principal, to be ergodic, although this is hindered by the required small step size.

**Fig. 2:**
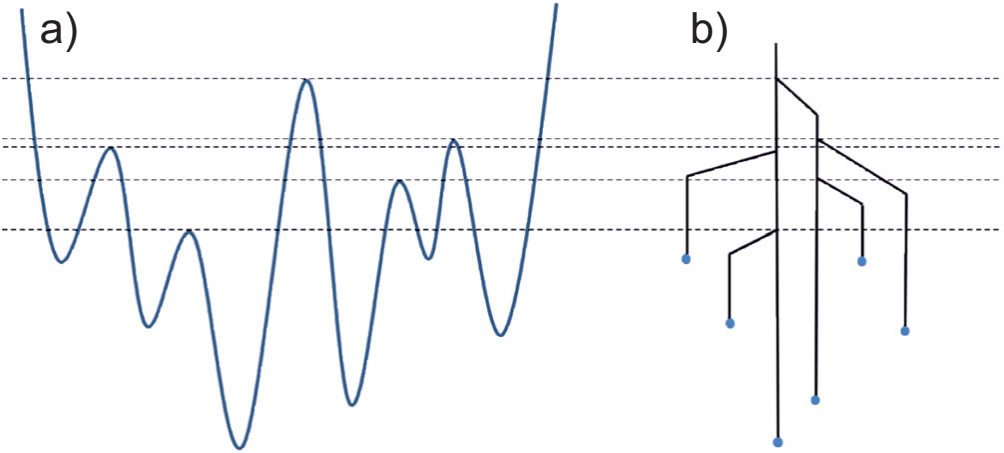
Disconnectivity graph. An example of tree-like representation (**b**) of an example energy landscape (**a**).

From a sequence of pockets sampled with increasing lid energies, a tree structure, often called a disconnectivity graph, can be constructed to represent the energy landscape^30–32^. The disconnectivity graph condenses the continuous, high-dimensional potential energy surface into the set of discrete local minima and information on the energy barriers that separate them. To construct the disconnectivity graph, the energy landscape is analysed at a set of energies along the vertical axis. The nodes at a given energy, *E*_*i*_, represent superbasins on the energy surface: the set of local minima that are connected by pathways below *E*_*i*_. Moving up in energy, nodes are connected as higher energy pathways connect groups of superbasins, while moving down on the graph leads to disconnections until the end of each branch, corresponding to single local energy minima. The horizontal axis has no direct physical meaning and is introduced for visualization, so that structures connected by lower energy barriers are grouped together. Vertices along the branches between nodes and minima are also for visualization only. A schematic of a one-dimensional potential energy surface and its associated disconnectivity graph is shown in Fig. 2.

In most simulations presented in this work, the lid energy was increased in increments of 5 kJ mol^−1^, which is chosen as a balance between precision in the calculated energy barriers and the need to explore a wide energy range. For each threshold simulation, the lid energy was increased from the minimized energy of the initial structure. Sampling was started from multiple initial structures, usually corresponding to the energy minimized versions of observed polymorphs, which leads to different energy grids from different start points. When plotting the disconnectivity, we used a new energy grid starting from the lowest energy among all the initial structures, with the increment of 5 kJ mol^−1^, to merge trajectories into one graph. Any lid energy from a single threshold algorithm was rounded up to the closest lid energy in the new grid.

The types of move allowed in the threshold simulations were molecular translations, rotations, perturbations of unit cell lengths and angles, as well as unit cell volume changes. Cutoffs on each move type (see ‘Methods’) were chosen to give similar energy changes for different move types. Probabilities for each move type were set according to the proportion of the total number of degrees of freedom for each move type, in the same way as in our implementation of basin hopping for CSP^33^. The sampling at each lid energy and total lengths of simulations differs between systems, depending on the number of degrees of freedom and energy window that needed to be covered. In the current work, a fixed number of steps was performed at each lid energy. An adaptive schedule was investigated (see Supplementary Methods) but due to the difficulty of choosing the convergence criteria, it did not improve the completeness of sampling. With energy minimization performed on all accepted steps (see ‘Methods’), and similar numbers of accepted steps to the numbers of structures generated during CSP searches, the computational cost involved in performing the threshold simulations is similar to the cost of performing CSP for the molecules studied here.

We have studied rigid molecules in this work, so molecular geometries were constrained to their optimized geometries from density functional theory (DFT) calculations. Lattice energy calculations were performed with the DMACRYS software^34^, using an empirically parametrized exp-6 intermolecular repulsion-dispersion potential with electrostatics described by atomic multipoles calculated from the DFT electron distribution (see ‘Methods’). Extending the method to molecules with conformational flexibility will require two major modifications: the inclusion of intramolecular degrees of freedom in the Monte Carlo steps and the use of an energy model that treats inter- and intra-molecular interactions during energy minimisation. Cutoffs on intramolecular distortions will need to be determined based on the typical energy changes resulting from different types of intramolecular perturbations.

To put more emphasis on the connections between low-energy structures, the disconnectivity graph is not presented for the whole energy range. The highest energy barrier between initial structures is taken as the upper limit and any local minima connected at lid energies higher than this upper limit are not presented on the disconnectivity graph. The disconnectivity graphs over the entire sampled energy range are presented in the Supplementary Notes.

For comparison to the energy landscape generated from the threshold algorithm, CSP was performed for each molecule using quasi-random sampling (see ‘Methods’).

### Sampling within a space group

We start with two examples where polymorphs exist with the same space group symmetry, so that a transformation between their corresponding local energy minima should be possible with space group symmetry constrained, i.e. Monte Carlo moves are only allowed which maintain the original symmetry. This is performed by perturbing the asymmetric unit of the crystal structure and applying symmetry-related perturbations to all other molecules in the unit cell. Constraints are also applied to unit cell parameters, where these are required to maintain space group symmetry.

The connections between structures found in this way exclude pathways that break symmetry, which might be lower in energy. However, the symmetry constraints simplify the simulation and we examine the picture of the landscape that we obtain with these constraints.

#### Indigo

**Fig. 3:**
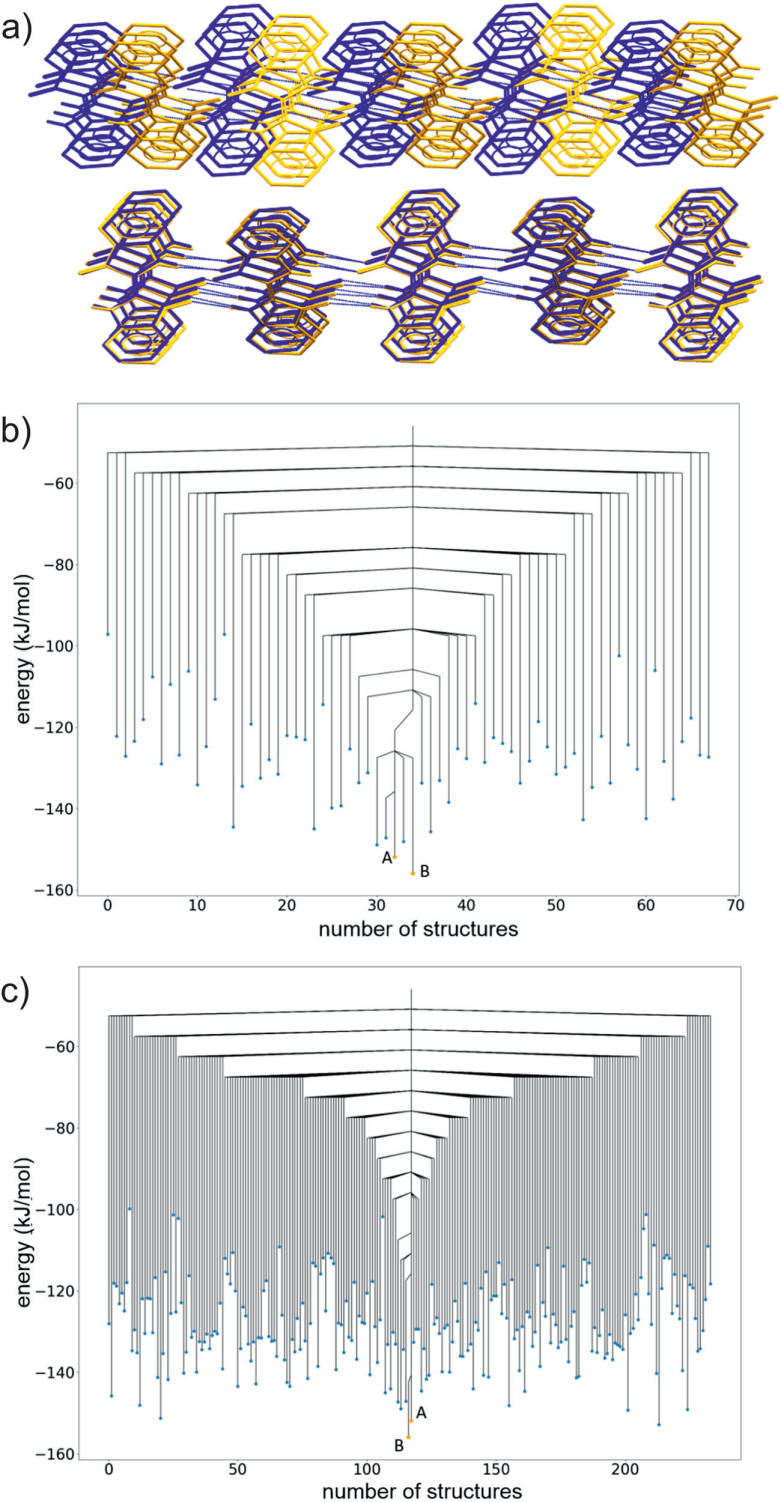
Structures and disconnectivity graphs for indigo. **a** Overlay of the packing of polymorphs A (blue) and B (orange) of indigo. Intermolecular hydrogen bonds are shown as blue dashed lines. Non-hydrogen bonding hydrogen atoms are hidden. **b**, **c** Disconnectivity graph from threshold simulations for indigo space group **b** *P*2_1_ and **c** *P*2_1_/*c*. Blue points are minimised structures. Orange points represent the initial structures. The horizontal axis labels the number of structures located during the threshold simulation. The number of structures differs between (**b**) and (**c**) due to the different number of structures sampled in the two space groups. The order of structures is chosen to group together structures separated by low energy barriers. A disconnectivity graph showing the entire sampled energy range is shown in Supplementary Fig. 2.

Indigo (Fig. 1a) is known to form two polymorphs^35,36^, named *A* and *B*, both containing layers of hydrogen-bonded molecules. The structure of these layers is almost unchanged between polymorphs *A* and *B*, but their structures differ in the arrangement of these layers (see overlay, Fig. 3a). Thus, the lowest energy pathway between polymorphs should not disrupt the hydrogen bonding and is expected to involve a relatively low energy barrier.

Both polymorphs have space group symmetry *P*2_1_/*c* with half a molecule in the asymmetric unit of the unit cell (both molecules lying on centres of inversion) and thus two molecules in the unit cell. To allow free molecular translations, unconstrained by the position of crystallographic inversion centres, the most symmetric representation used for simulation of these structures was *P*2_1_ with whole molecules in the asymmetric unit. Crystal structure prediction in *P*2_1_ finds polymorphs *A* and *B* as the two lowest energy crystal structures (see Supplementary Fig. 1), with *B* having a calculated lattice energy 4.0 kJ mol^−1^ below *A*. For comparison, the two polymorphs are reported to be nearly equi-energetic when evaluated using periodic DFT with a plane wave basis set and many-body dispersion correction^37^.

Monte Carlo simulations were started from the CSP structures matching both polymorphs, which were continued for 10 lid energies, incremented by 5.0 kJ mol^−1^ with 1000 attempted steps under each lid (covering a total 50.0 kJ mol^−1^ energy window). From threshold simulations in space group *P*2_1_, the first connection between polymorphs *A* and *B* is found when the lid is 26.0 kJ mol^−1^ above *A* (30.0 kJ mol^−1^ above *B*) (Fig. 3b). Below this energy, no other structures are connected to *B*, one slightly higher energy structure is connected to *A* and two additional structures connect to *A* and *B* at the same lid energy. All three of the additional crystal structures within the basin connected to *A* and *B* maintain the same hydrogen bond motif, but with greater differences in molecular orientation around the hydrogen bonds than between polymorphs *A* and *B*. No further structures are connected to the basin containing these five local energy minima (*A*, *B* and the three higher energy structures) until the lid energy is raised a further 15 kJ mol^−1^. Globally, the results give a picture of an energy landscape that is funneled towards a small set of structures, all featuring the same favoured hydrogen bonding motif, with the two known polymorphs as the lowest energy local minima within this energy superbasin.

To investigate the effect of performing the threshold Monte Carlo simulation with different symmetry constraints, simulations were also performed with larger unit cells, containing four molecules in space group *P*2_1_/*c*. Both known polymorphs can also be described with this symmetry. The resulting disconnectivity graph is shown in Fig. 3c. The overall picture of a single funnel towards polymorphs *A* and *B* is maintained, but a notably lower energy transition is found between the known polymorphs, when the lid is 11.0 kJ mol^−1^ above *A*, 15.0 kJ mol^−1^ above *B*. As a result, the connection between *A* and *B* occurs at a lower energy than their connection to any other local minimum on the landscape. While both indigo simulations yield useful information on the structure of the crystal energy landscape, the results confirm our expectation that the symmetry constraints applied during sampling can have an important influence on the details of the disconnectivity graph.

The 5 kJ mol^−1^ increments in threshold energy limit the precision with which the energy barrier between the two polymorphs can be estimated. Knowing that the transition occurs when the threshold is 15 kJ mol^−1^ or less above polymorph *B*, we re-sampled the landscape in space group *P*2_1_/*c* (four molecules in the unit cell), with smaller threshold increments of 1 kJ mol^−1^ and 1000 steps under each lid energy (5 × more sampling per kJ mol^−1^ increase in the lid energy). Again, simulations were started from both polymorphs, for 15 increases in the lid energy. With this increased sampling, the energy barrier is now located when the threshold is 10 kJ mol^−1^ above polymorph *B*, 6 kJ mol^−1^ above *A*, and no other local energy minima are visited up to the highest energy threshold.

The negligible change with smaller lid energy steps and increased sampling gives us confidence that we have sampled the landscape sufficiently. The results illustrate a strategy that can be used to explore energy landscapes: an initial simulation with large energy threshold increments to capture the global structure of the energy landscape, followed by targeted re-sampling using smaller steps to refine the results in important regions of the energy landscape.

#### 1:1 Nicotinamide:benzoic acid co-crystal

As a second example, we studied the 1:1 nicotinamide:benzoic acid (GAZCES) co-crystal as a system with more degrees of freedom (due to two molecules in the asymmetric unit), but where the known polymorphs again have the same space group symmetry. Nicotinamide:benzoic acid is a highly polymorphic co-crystal; four polymorphs have been observed under mechanochemical co-crystallization conditions, but only polymorphs **I** and **II** have had their structures determined^28^. Both characterized polymorphs are in space group *P*2_1_/*c*.

**Fig. 4:**
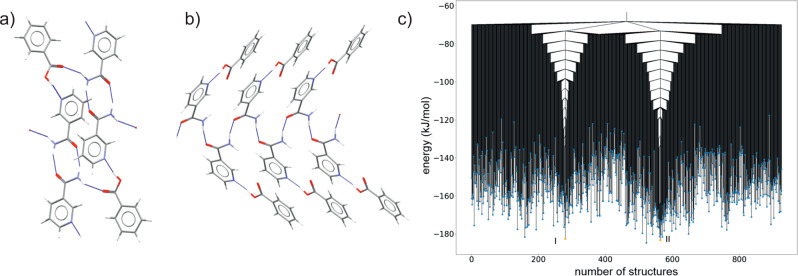
Structures and disconnectivity graph of the nicotinamide:benzoic acid co-crystal. Hydrogen bonding in polymorphs **a** **I** and **b** **II** of the nicotinamide:benzoic acid co-crystal. Carbon atoms are grey, oxygen red, nitrogen blue and hydrogen white. Hydrogen bonds are shown as dashed blue lines. **c** Disconnectivity graph from threshold simulations for the 1:1 nicotinamide:benzoic acid co-crystal in space group *P*2_1_/*c*. The horizontal axis labels the number of structures located during the threshold simulation. The order of structures is chosen to group together structures separated by low energy barriers. A disconnectivity graph showing the entire sampled energy range is shown in Supplementary Fig. 4.

Unlike indigo, the polymorphs of this co-crystal differ in their hydrogen bonding (Fig. 4): polymorph **I** has nicotinamide doubly hydrogen-bonded dimers connected by hydrogen bonds to benzoic acid molecules, while polymorph **II** contains an extended hydrogen-bonded nicotinamide chain with benzoic acid hydrogen bonding to the nicotinamide pyridyl nitrogen atoms at the edges of these chains.

Threshold simulations were started from the structures corresponding to **I** and **II** from the CSP simulations. **I** and **II** have very similar calculated lattice energies (**I** has a calculated lattice energy 0.6 kJ mol^−1^ below **II**) and are located in the low energy region of the landscape (Supplementary Fig. 3). Due to the greater complexity of the landscape, 3000 steps were performed at each value of the lid energy, which was increased in 5.0 kJ mol^−1^ steps up to 150.0 kJ mol^−1^ above the initial structures, i.e., 30 increases in the threshold energy and a total of 90,000 attempted perturbations from each starting structure.

The threshold simulations reveal a dual-funneled energy landscape with deep basins centred on polymorphs **I** and **II** (Fig. 4c). Polymorph **I** is the lowest energy structure in its funnel (left of Fig. 4c), while one lower energy structure is located in the funnel containing **II** (right of Fig. 4c). Several lower energy structures are located by CSP in space group *P*2_1_/*c*, but not sampled during the threshold simulations; these structures might lie outside of the two funnels, where little sampling has been performed. A complete picture of the energy landscape would require threshold sampling from some of the unobserved polymorphs as well as **I** and **II**.

The lowest energy connection between the funnels is approximately 120 kJ mol^−1^ above **I** and **II**. Thus, the threshold simulation is able to locate a pathway connecting these two very different crystal structures and the rearrangement in hydrogen bonding required to transform between them results in a high energy barrier. A lower energy connection between **I** and **II** might be found if space group symmetry constraints were removed from the Monte Carlo sampling, but it is unlikely that the global structure of the landscape would be changed. The funneled landscape gives an impression that polymorph selection will be strongly influenced by crystallization conditions, which could lead crystal growth into one funnel or the other, and that, once grown, interconversion of the polymorphs is unlikely without a large energetic input. Indeed, both **I** and **II** are reported to be stable for months once isolated^28^.

### Sampling between space groups in a *P*1 cell

Simulations of the indigo and co-crystal examples were simplified by their polymorphs having the same space group symmetry. However, in practice, transformations between structures have no symmetry constraints. Therefore, we expect to obtain a better estimation of the actual energy barrier between structures by sampling with as many constraints as can practically be removed from the simulation. We also want to be able to analyse energy landscapes involving crystal structures with different symmetries. Here, we look at two examples involving known crystal structures with different space group symmetry. To remove the symmetry constraints, we use *P*1 unit cells. In the following examples, we construct *P*1 unit cells containing sufficient molecules to be able to represent all the known polymorph crystal structures, i.e., the lowest common multiple of *Z* (the number of formula units in the unit cell) for all known structures. Thus, the simulations presented below are not fully unconstrained—translational symmetry is imposed by the unit cell—but the models have sufficient flexibility to describe the polymorphs of interest. The approach could be extended to be able to visit more possible packing symmetries by expanding the unit cell to contain more molecules.

#### Tetrolic acid

**Fig. 5:**
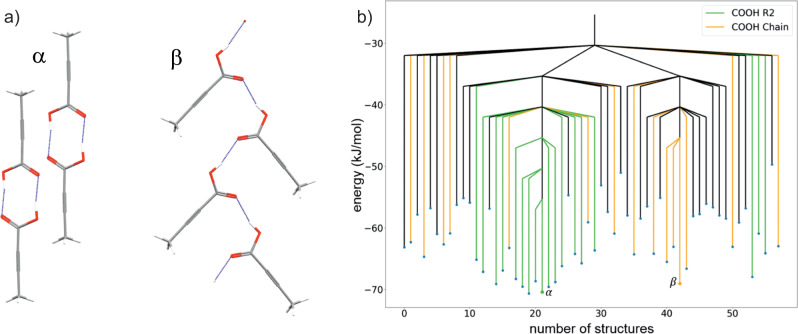
Hydrogen bonding and disconnectivity graph for tetrolic acid. **a** Hydrogen bonding in the two known polymorphs of tetrolic acid. Carbon atoms are grey, oxygen red and hydrogen white. Hydrogen bonds are shown as dashed blue lines. **b** Disconnectivity graph from threshold simulations for tetrolic acid with two molecules in a *P*1 unit cell. The horizontal axis labels the number of structures located during the threshold simulation. The order of structures is chosen to group together structures separated by low energy barriers. A disconnectivity graph showing the entire sampled energy range is shown in Supplementary Fig. 5. Colours of edges represent the hydrogen bond motif of connected minimized structures where black edges are structures identified as having neither of the motifs. Hydrogen bond motifs were identified with Mercury^59^, identifying hydrogen bonds as H...O contacts with a separation less than the sum of van der Waals radii +0.1 Å and an O–H...O angle >125^∘^.

Tetrolic acid has two known polymorphic forms: a triclinic structure in space group $P\overline{1}$ and a monoclinic structure in space group *P*2_1_^38^, referred to as *α* and *β*, respectively. The two structures have different hydrogen bond motifs: the carboxylic acid groups form cyclic, doubly hydrogen-bonded dimers in *α* and infinite hydrogen bond chains in *β* (Fig. 5a). Both crystal structures have two molecules in the unit cell, so simulations were performed using *P*1 unit cells containing two molecules. The number of degrees of freedom to sample this system was the same as the nicotinamide:benzoic acid co-crystal, so we applied the same number of Monte Carlo steps (3000) at each lid energy, which was raised in 5 kJ mol^−1^ increments, starting simulations from the structures of *α* and *β*.

An interesting detail for tetrolic acid is that the molecular geometry is chiral: the molecule and its mirror image differ by rotation of the methyl group. This is important for the interconversion of the two polymorphs because space group $P\overline{1}$ contains an inversion centre while space group *P*2_1_ does not. Therefore, the unit cell of the *α* polymorph contains two molecules with different methyl hydrogen orientations, while *β* contains two copies with the same geometry. In the rigid-molecule approach used here, structures sampled from these two starting points cannot agree perfectly. However, because the difference in methyl hydrogen positions has very little impact on intermolecular interactions, we find pseudo-$P\overline{1}$ structures in the simulation started from *β* and pseudo-*P*2_1_ structures in the simulation started from *α*. Thus, the structure matching methods, which ignore hydrogen atom positions, find matching structures in the two trajectories and we can find connections between the structures. In the more general case, the rigid-molecule constraint must be removed and intramolecular perturbations included in the simulations, to allow interconversion between chiral molecular conformations.

Threshold simulations reveal a similar energy landscape structure as found for the GAZCES co-crystal, with separate funnels containing structures *α* and *β* (Fig. 5b). Because the simulations were run without space group constraints, a pathway could be found between the polymorphs. This connection was located when the lid energy was 40 kJ mol^−1^ above *α*, which has the slightly (1.4 kJ mol^−1^) lower calculated lattice energy of the two known polymorphs. The relatively high energy barrier is unsurprising, considering the breaking of hydrogen bonds required to transform between *α* and *β*, along with substantial reorientation of the molecules.

To confirm that the structure of the landscape is determined largely by the hydrogen bond interactions, the local minima within each funnel were classified by their hydrogen bond motifs (Fig. 5b). Indeed, all structures connected to *α* or *β* by barriers lower than 25 kJ mol^−1^ maintain the same hydrogen bonding as the starting structure, i.e. dimers within the *α* funnel and chains within the *β* funnel. At thresholds 30 and 35 kJ mol^−1^ above *α*, we start to see changes in hydrogen bond motifs: two structures with hydrogen bond chains within the *α* funnel and several crystal structures within both funnels that are not classified as either chains or dimers. Finally, at 40 kJ mol^−1^ above *α*, the two funnels are connected.

Although there is a clear relationship between the structure of the disconnectivity graph and the strongest interactions between molecules, we cannot necessarily relate the transitions observed during the threshold simulations to physically realistic pathways. In particular, we found that some transitions occur via structures with large separations between layers of molecules (see Supplementary Methods). These pathways create space for large-scale rearrangement of molecules and might indicate the lack of reasonable single-crystal to single-crystal transition pathways that maintain translational symmetry between some pairs of structures.

**Fig. 6:**
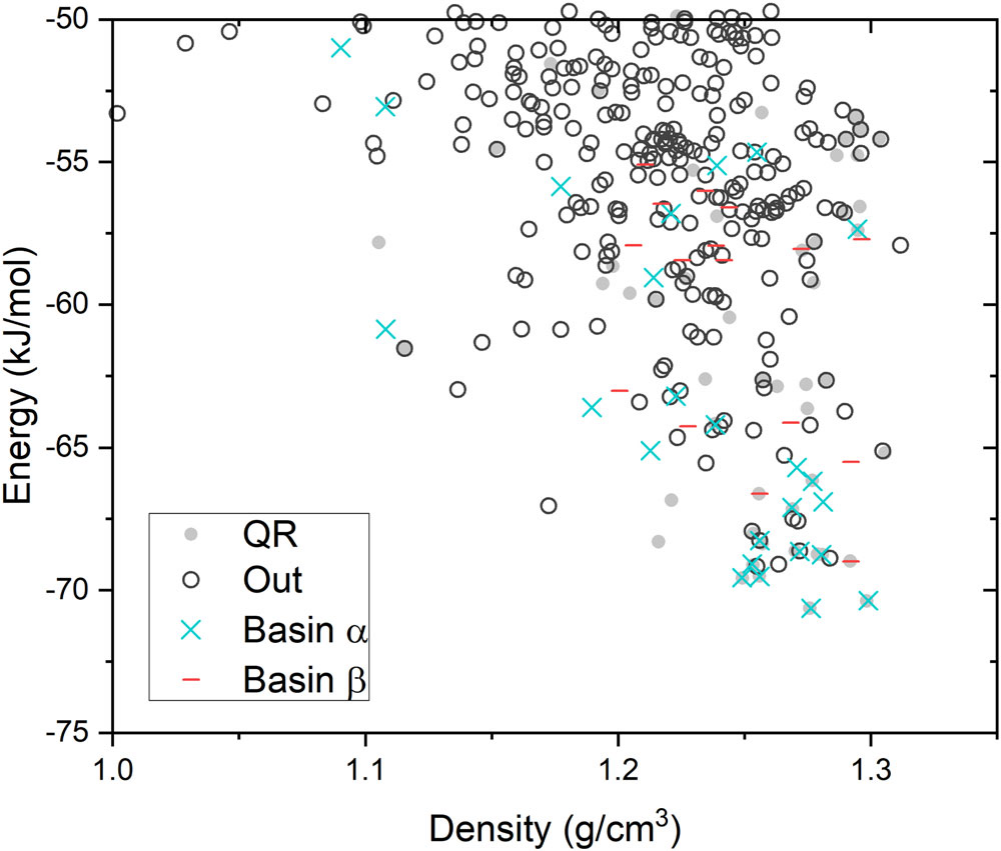
Lattice energy vs density plot of the landscape of predicted crystal structures of tetrolic acid. Local minima located after optimisation of structures from the threshold sampling are classified as belonging to either main basins (*α* and *β*) or not within one of these basins (black circles, labelled 'Out'). Crystal structure prediction results from quasi-random sampling (QR) are shown for comparison, with searches performed in space groups $P\overline{1}$ and *P*2_1_.

We now compare the results of the threshold sampling to the output from CSP on tetrolic acid, in this case restricting CSP to the space groups of the two known polymorphs ($P\overline{1}$ and *P*2_1_). The resulting structures are shown in Fig. 6 in the representation often used in CSP studies: plotting the energy vs density of all predicted structures. Structures sampled during threshold searches are also shown and labelled by whether they belong to the funnels around *α*, *β* or are disconnected from those superbasins at a lid energy 40 kJ mol^−1^ above *α*. A first observation is that crystal structures in the *α* and *β* funnels occupy overlapping regions of energy-density space, so that the traditional CSP energy-density representation does not convey the important information about which structures belong to connected regions of the high-dimensional energy landscape. The disconnectivity graph conveys this information more clearly.

We also find that some structures are found in the threshold search, but not CSP, and vice versa. The threshold search is able to locate structures that are not accessible in the CSP because of lower symmetry constraints: some structures do not belong to either space group included in CSP. On the other hand, structures found by CSP, but not the threshold search indicate that the threshold sampling has not fully explored the energy landscape. However, we note that overlap between QR and threshold structures is very good in the important low-energy region of the landscape.

#### TTBI

**Fig. 7:**
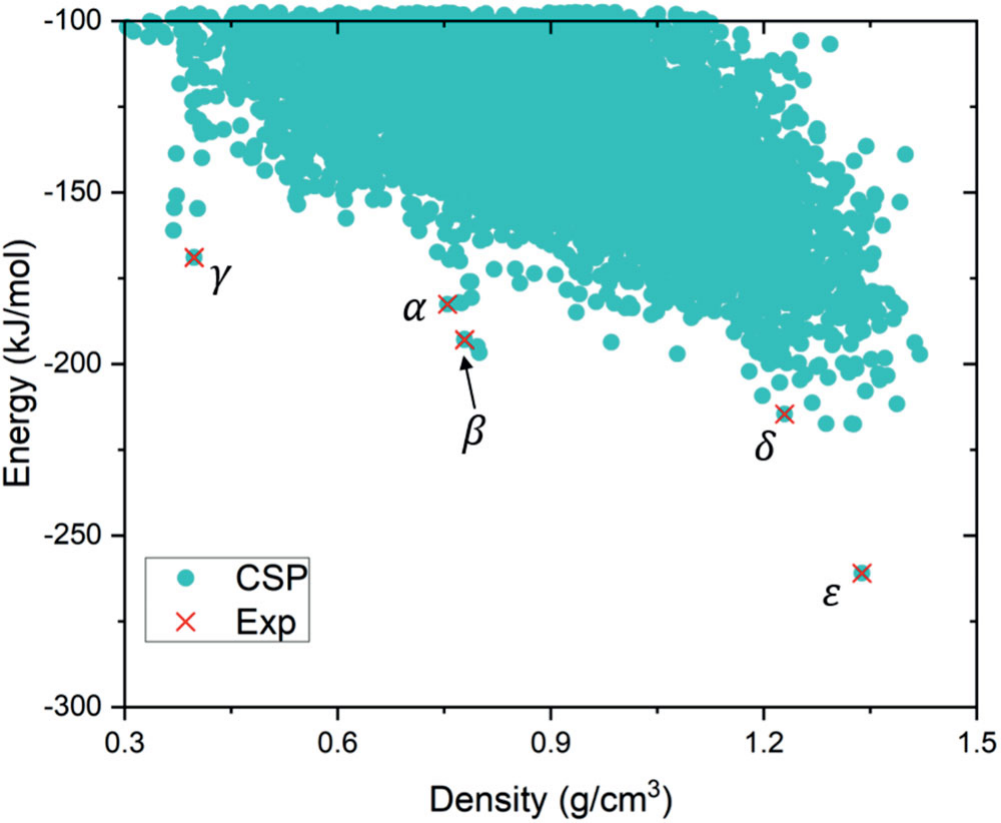
Lattice energy vs density plot of the landscape of predicted crystal structures of TTBI. Lattice energy vs density plot of the landscape of predicted crystal structures of TTBI from CSP (quasi-random sampling), with predictions performed in *P*1 with four molecules in the unit cell. The experimentally observed structures are labelled *α*, *β*, *γ*, *δ* and *ε*. The energy landscapes generated from threshold simulations are presented in Supplementary Figs. 6, 7.

The final molecule investigated is TTBI (Fig. 1c), which has five known polymorphs^6,39,40^, four of which occupy high energy regions of the crystal structure landscape. The positions of the observed structures are indicated on the crystal energy landscape (Fig. 7). While the *ε* polymorph corresponds to the energetic global minimum, the other four observed structures fall outside the usual energetic range of polymorphism^41^. The proposed explanation for the observation that these very high energy structures can be isolated as stable materials is that they occupy deep, isolated regions of the energy landscape, which is hinted at by the ‘spikes’ of structures that fall below the bulk of structures on the energy-density landscape^6^*; α*, *β* and *γ* are low energy structures within these spikes. We apply the threshold algorithm to add information to the CSP landscape and improve our understanding of the observed polymorphism of TTBI.

**Fig. 8:**
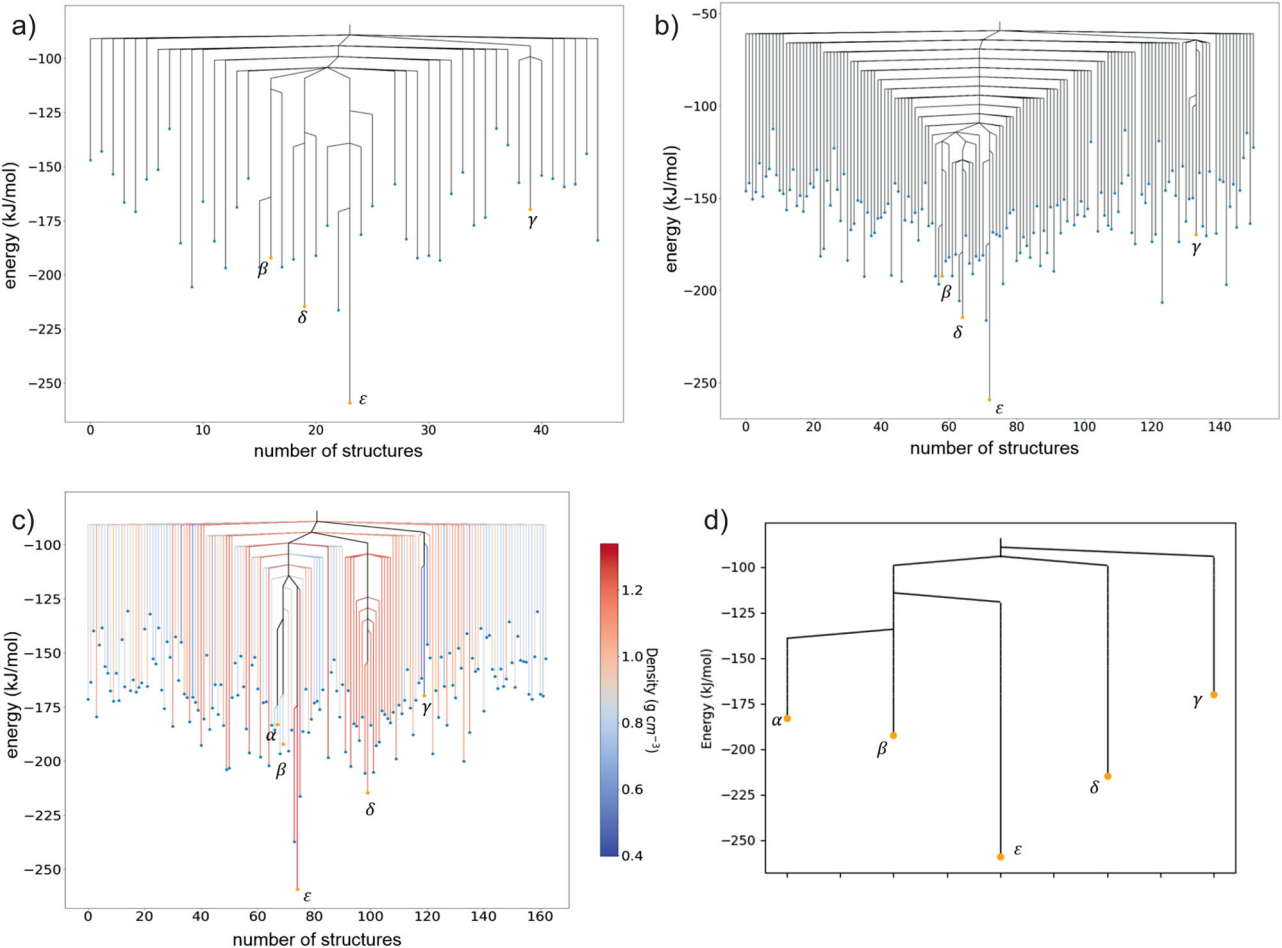
Disconnectivity graphs from threshold simulations for TTBI. Threshold results are shown from sampling in space groups: **a** $P\overline{1}$ (two molecules per unit cell), **b** *P*2_1_/*c* (four molecules per unit cell) and **c** *P*1 with four independent molecules per unit cell. **d** A simplified graph from the simulation in *P*1 with all local minima apart from the five landmark structures (*α*, *β*, *δ*, *γ* and *ε*) hidden. The branches in (**c**) are coloured to show the crystal density of each structure. The horizontal axis labels the number of structures located during the threshold simulation. The order of structures is chosen to group together structures separated by low energy barriers. Disconnectivity graphs showing the entire sampled energy range are shown in Supplementary Figs. 8, 9.

Of the five experimentally observed TTBI structures, four (all but *α*) have space group symmetries that could be sampled in either space group $P\overline{1}$ or *P*2_1_/*c* with one independent molecule (${Z}^{\prime}=1$). The results of threshold simulations performed in $P\overline{1}$ and *P*2_1_/*c*, starting trajectories from *β*, *γ*, *δ* and *ε*, are shown in Fig. 8a, b. All searches used 5 kJ mol^−1^ increments in the lid energy and 1000 steps were attempted at each lid energy.

The threshold simulations in $P\overline{1}$ and *P*2_1_/*c* yield disconnectivity graphs with similar structures and high energy barriers between the initial structures (lid energies at which transitions are found are summarised in Table 1). The connectivity structure shows a deep energy basin centred on *ε* with very few connected local minima until the energy lid is increased to very high energies. In the $P\overline{1}$ simulation, *β*, *δ* and *ε* are all connected at the same lid energy, +155 kJ mol^−1^ above *ε* (+110 kJ mol^−1^ above *δ* and 67 kJ mol^−1^ above *β*). The results are very similar in *P*2_1_/*c*. As with the indigo polymorphs, there is a slight lowering of the lid energies at which several of the transitions occur in this larger unit cell: the connection between *β* and *δ* is lowered by 10 kJ mol^−1^ compared to the $P\overline{1}$ results, while their connection to *ε* is lowered by only 5 kJ mol^−1^.

**Table 1:** Lid energies at which transitions are located between *γ*, *β*, *δ* and *ε* in threshold sampling in space groups $P\overline{1}$ (upper right entries) and *P*2_1_/*c* (lower left entries).

	*ε*	*δ*	*β*	*γ*
*ε*	*−259.2*	−104.2	−104.2	−89.2
*δ*	−109.2	*−214.5*	−104.2	−89.2
*β*	−109.2	−114.2	*−192.1*	−89.2
*γ*	−59.2	−59.2	−59.2	*−169.7*

Energies in italics along the diagonal are the calculated lattice energies of the four structures. All energies are in kJ mol^−1^.

The basin containing the low-density *γ* polymorph is separated by an even larger energy barrier from *β*, *δ* and *ε*. This energy barrier connecting the *γ* trajectory to the other three structures shows the largest difference between results in the two space groups. The lid energy at which *γ* connects to the others is −89.2 and −59.2 kJ mol^−1^ in $P\overline{1}$ and *P*2_1_/*c*, respectively, representing a barrier of 80 or 110 kJ mol^−1^ for the transformation of *γ* into the global energy minimum *ε* or one of the other known polymorphs. Despite the large quantitative difference found when sampling with different symmetry constraints, the same qualitative picture emerges: globally, *γ* is separated by a high energy barrier from the main pocket formed by *ε*, *β* and *δ*, occupying its own funnel on the connectivity graph. This finding agrees with the surprisingly good reported thermal stability of the *γ* polymorph, despite its exceptionally low density^6^.

The results shed light on the stability of the *δ* polymorph. Although *δ* does not occupy a clear spike on the energy-density representation of the landscape (Fig. 7), the threshold simulations demonstrate that this structure does occupy a deep energy basin with high energy barriers preventing transformation to lower energy structures.

The *α* polymorph of TTBI crystallizes in space group *P*4_2_/*m* with four molecules in the unit cell^39^ and cannot be represented in the unit cells included in the $P\overline{1}$ and *P*2_1_/*c* simulations. To include *α*, we performed a further set of simulations in a *P*1 unit cell containing four symmetry-independent molecules, starting trajectories from all five structures (*α*, *β*, *γ*, *δ*, *ε*) and attempting 5000 steps per lid energy. Due to the wide range of initial energies, the number of lids varied between trajectories, with the longest simulation started from *ε* involving 250,000 total Monte Carlo steps.

The resulting disconnectivity graph from the *P*1 threshold simulation is shown in Fig. 8c, where the branches corresponding to each local energy minimum are coloured by the density of the corresponding crystal structure. Apart from now including *α*, a difference with respect to the $P\overline{1}$ and *P*2_1_/*c* results is that the lower symmetry allows more distinct local minima to be identified within the superbasins corresponding to the known polymorphs; this is particularly noticeable around *δ*, where 17 structures are connected to *δ* at lower energy barriers than the connection of the *δ* superbasin to those of *ε*, *α* and *β*. To more clearly visualise the relationships between the five polymorphs, a simplified disconnectivity graph is shown in Fig. 8d, in which all structures other than the five starting structures are hidden. The lid energies at which transitions are found between the five structures are summarised in Table 2.

**Table 2:** Lid energies at which transitions are located between *γ*, *α*, *β*, *δ* and *ε* in threshold sampling in space group *P*1 with four independent molecules in the unit cell (upper right entries).

	*ε*	*δ*	*β*	*α*	*γ*
*ε*	*−259.2*	−94.2	−114.2	−114.2	−89.2
*δ*	−109.2	*−214.5*	−94.2	−94.2	−89.2
*β*	−109.2	−114.2	*−192.1*	−134.2	−89.2
*α*	–	–	–	*−183.1*	−89.2
*γ*	−89.2	−89.2	−89.2	–	*−169.7*

The entries in the lower left are the lowest energy lid at which transitions are found in the simulations in *P*2_1_/*c* and $P\overline{1}$. Energies in italics along the diagonal are the calculated lattice energies of the four structures. All energies are in kJ mol^−1^.

As with the results from the higher symmetry simulations, we find the barrier separating the *γ* polymorph from other observed forms to be the highest; the connection between *γ* and the other polymorphs is found at the same lid energy in the *P*1 simulation as the simulation with $P\overline{1}$ symmetry constraints in the smaller unit cell. This fulfills our expectation that sampling in *P*1 should find a transition path with an energy barrier equal to or lower than that found in space group-constrained trajectories.

The pair of polymorphs related by the lowest energy barrier is *α* and *β*, where the lid energy at which their trajectories meet is just under 50 kJ mol^−1^ above the calculated energy of *α*. The result is in line with the experimental observation that a transformation occurs between the *α* and *β* polymorphs^6^, as well as molecular dynamics studies^6^ in which the structures of all other observed TTBI polymorphs showed only fluctuations about their known crystal structures at 300 K, apart from *α*, which partially transformed to *β* in a short, 500 ps, simulation. These structures also occupy the same ’spike’ on the energy-density representation of the crystal structure landscape. We note that the disconnectivity graph tends to group structures of similar density; the lowest energy barriers tend to be found between structures that are close in density.

### Energy landscape featuring

The disconnectivity graph that is generated from the threshold simulations could be viewed as a form of clustering of local energy minima, grouping those that are related by the lowest energy barriers. The results for tetrolic acid demonstrate that there is a link between the basin structure on the crystal energy landscape and geometric features of the crystal structures—in this case, hydrogen bonding motif. The results for TTBI also suggest that crystal density differences explain some of the grouping of local energy minima into the superbasins on the energy landscape. Therefore, we asked the question whether structural descriptors commonly used in machine learning applications could provide a more general geometrical descriptor of structural similarity that correlates with the clustering of local minima based on heights of energy barriers. If so, this could improve our physical understanding of the information gained by applying geometrical descriptors to CSP landscapes, which has started to gain attention for identifying families of related structures, with the goal of discovering structure-property relationships^42–44^. Furthermore, a successful geometrical clustering could replace the computationally expensive local energy minimization procedure to identify the basin to which each point on the Monte Carlo trajectory belongs.

We have taken the GAZCES co-crystal energy landscape in space group *P*2_1_/*c* as an example. This system was chosen for investigation due to the clear structure of the energy landscape and the sufficiently large number of structures in each basin from threshold simulations. For analysis, the total energy landscape was divided into three regions: two funnels, each containing an initial structure (polymorph **I** or **II**) and the structures outside the two basins. Two common descriptors for crystal systems—smooth overlap of atomic positions (SOAP)^45^ and atom-centred symmetry functions (ACSFs)^46^—were applied with common data featuring methods.

**Fig. 9:**
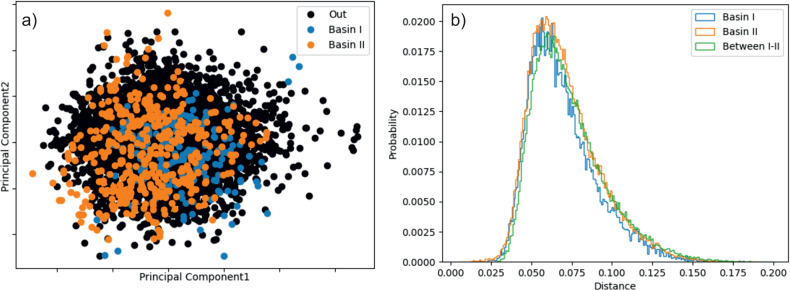
PCA of the nicotinamide:benzoic acid co-crystal predicted structures. **a** The first two principle components of the SOAP kernel for structures for co-crystal GAZCES in space group *P*2_1_/*c*, coloured according to the basin identified from threshold simulations. Structures labelled in black fall outside of the two main basins (see Fig. 4c). **b** Distribution of pairwise dissimilarities by the SOAP REMatch kernel between and within energy basins **I** and **II**.

We first investigated dimensionality reduction using PCA of the ACSFs and the SOAP kernel. In both cases, the descriptors were flattened over all atoms and, for symmetry functions, radial and angular functions were merged into one vector. With either descriptor, the eigenvalues corresponding to the first two principle components had twice the magnitude of the third. We plot the structures onto these first two principal components in Fig. 9a (see Supplementary Fig. 10 for the corresponding plot using ACSFs). In neither case is there clear differentiation of structures corresponding to basins **I** and **II**, as identified by the threshold algorithm; the structures from both basins, and those outside of the two basins, overlap in this projection onto the first two principal components.

In case the overlap of basin **I** and basin **II** structures seen in the PCA visualization is due to the dimensional reduction, we also tested density-based clustering in the original, high-dimensional descriptor space. The HDBSCAN* algorithm^47^ was applied to the same dataset (the GAZCES co-crystal set of local energy minima in *P*2_1_/*c*). However, trying different minimum cluster sizes and, for ACSFs, different measures of the pairwise distance between structures (see Supplementary Figs. 11–14), no clustering could be obtained that aligns with the grouping of structures from the threshold simulation results. To understand these results, we examined the distributions of distances between structures within each basin and between basins. The distribution of distances between structures was found to be similar within and between energy basins (Fig. 9b and Supplementary Figs. 11–14), indicating that geometrical similarity based on these atom-centred geometrical descriptors is not necessarily a good indicator of structures that are ’closer’, in terms of energetic accessibility, on the energy landscape.

Similar results were observed for tetrolic acid (see Supplementary Figs. 15–18): descriptors of local atomic environment do not effectively distinguish between structures belonging to either superbasin identified during the threshold simulations.

## Conclusions

We have implemented the threshold algorithm to study the energy landscapes of molecular crystal structures with a force field energy model using atomic multipole electrostatics. The method has been applied to four molecular organic crystal systems to examine the energy barriers between known polymorphs and the global structure of their crystal energy landscapes, which we visualize using disconnectivity graphs.

The structures of the energy landscapes vary between the systems studied here, from a single funnel for the crystal structures of indigo, where hydrogen bonding is conserved between polymorphs, to multiple energy funnels when polymorphs differ in the arrangement of their strong intermolecular interactions. Thus, the threshold simulation results reinforce our chemical intuition and provide a quantification of the differences in energy barriers between different polymorphic systems.

Although the structures of the energy landscapes for the molecules studied here can be rationalized in terms of intermolecular interactions, we find that for two systems where threshold simulations show clearly separate funnels on the energy landscape that the grouping of crystal structures in energy basins is not reproduced by clustering or dimensionality reduction based on commonly-used structural descriptors. Thus, the results are complementary to unsupervised machine learning approaches that have been applied to the analysis of crystal structure landscapes.

The influence of space group constraints on energy barriers has been examined; for the systems studied here, the qualitative global picture of the energy landscape is not strongly influenced by imposing symmetry constraints, but the magnitude of energy barriers is affected. Therefore, space group-constrained simulations can be used to gain initial insight into crystal energy landscapes, but more computationally demanding, unconstrained simulations are the best route for more quantitative results.

Our implementation of the threshold algorithm is currently limited to rigid molecules and, thus, only requires evaluation of intermolecular interactions. The extension to flexible molecules would be needed to apply the method to systems where changes in molecular conformation between polymorphs are important. The extension should be straightforward and would require perturbations that involve intramolecular distorsion, and an energy model that includes the inter- and intra-molecular energy contributions.

We believe that the method presented here is a powerful tool that can be applied in different situations to enhance our understanding of polymorphism. The work presented here has focussed on known polymorphs, where their structures provide the starting points for threshold simulations, which reveal the energetic relationship between the polymorphic structures. More generally, the method could be combined with crystal structure prediction methods without prior knowledge of the observed crystal structures. In this situation, all low energy structures, along with other landmark structures on the predicted energy landscape, could provide starting points and the threshold simulations would produce a global picture of the energy landscape and the molecule’s predicted polymorphs.

## Methods

### Monte Carlo perturbation

Five types of perturbation are implemented, including molecular translation and rotation, unit cell length, unit cell angle as well as volume expansion and contraction. The direction of molecular translation is defined by a random unit vector generated from a uniform distribution. The implementation of rotation first involves the generation of a random unit vector as rotation axis as well as the rotation angle to be rotated around that axis from a uniform distribution; these are then turned into a quaternion and applied to the target molecule. For lattice parameter perturbations, a flexible parameter is chosen whose value is changed directly. As to volume expansion and contraction, the volume fractional change is calculated as the volume after perturbation dividing the original volume and the coordinates of molecules in the unit cell are scaled with the cubic root of the volume fractional change.

In threshold simulations, the step size of each perturbation is taken from a uniform distribution among the range within the corresponding cutoff, except for the unit cell angles. Only unit cell angles ranging from 45 to 135 degrees are taken to be valid, other than trigonal crystal systems where the upper limit is 120 degrees, and are more likely to be changed to 90 degrees to avoid the problematic local minimization caused by a flat unit cell. Added with a shift based on how much it diverges from 90 degrees, the unit cell angle follows a symmetric triangular distribution with a peak located at 90 degrees. This perturbation surely breaks the detailed balance, which is acceptable since the purpose is structure prediction rather than ensemble sampling.

The following cutoffs were used as maximum perturbations in an attempted Monte Carlo move during threshold simulations: 0.50 Å for molecular translations; 0.05 radians for molecular rotations; 0.50 Å for unit cell length perturbations; 0.50 degrees for unit cell angle perturbations and 25 Å^3^ for unit cell volume changes. These were chosen to given similar distributions of energy changes for each type of perturbation. The cutoff of the volume change depended on the number of molecules in primitive unit cell, *Z*. The more molecules in the unit cell, the more pairs of interaction are affected by a volume change, and thus, the greater the energy changed. Therefore the actual maximum change of volume is *Z* multiplied by the cutoff. The magnitudes of individual moves were calculated by multiplication of a random number ranging from 0 to 1 by the move cutoff.

### Quasi-random sampling

Crystal energy landscapes generated from CSP are compared with results from threshold simulations as a reference. The procedure and methods included are described in detail in our earlier paper^48^. The structure generation used quasi-random sampling where a low-discrepancy sequence generated by Sobol method^49^ is mapped to the molecular position, orientation and lattice parameters not constrained by space group symmetry. Due to the use of the Buckingham potential, the interaction between two close atoms is nonphysical and such structures must be modified or removed before local lattice energy minimization. The idea used here is to detect whether two molecules are overlapping and expand the unit cell to remove the collision (if any). The convex hull^50^ is calculated for each molecule as its physically occupied space, and the separating axis theorem^51^ is used to expand the unit cell when two convex hulls overlap. The generated structure is lattice energy optimized to its local minimum with the molecule held rigid at its DFT optimized geometry. The energy calculation is the same as used in the threshold algorithm, described below.

Quasi-random structure generation was continued until 10,000 structures were successfully lattice energy minimized in each space group, for single-component crystal structures. For the co-crystal, the search was extended to 100,000 structures, and CSP with four independent molecules ($Z^{\prime} =4$) in *P*1 was continued until 1,000,000 crystal structures were successfully energy minimized.

### Lattice energy calculations

Lattice energy minimizations were performed with an anisotropic atom-atom force field model for intermolecular interactions, using the DMACRYS software^34^. Space group symmetry was constrained throughout all optimizations, apart from where we specify that calculations were performed in *P*1. We make the rigid-molecule approximation in this work, which assumes that the geometry of a molecule is unaffected by intermolecular interactions when forming a crystal. All molecular geometries were optimized using DFT at the B3LYP/6-311G** level of theory, starting from the geometries extracted from structures in the Cambridge Structural Database^52^. All DFT calculations were performed within the Gaussian09 software^53^.

Repulsion-dispersion interactions were modelled with the empirically parameterized FIT *exp-6* force field^54^. Electrostatic interactions were described with a distributed multipole electrostatic model based on the molecular charge densities calculated from a distributed multipole analysis^55^ of the B3LYP/6-311G** electron density, with multipole rank up to hexadecapole on each atom. Thus, the intermolecular atom-atom potential function has the form:

1 $${E}_{{{{{{\rm{lattice}}}}}}}=\frac{1}{2}\mathop{\sum}\limits_{M,N}\mathop{\sum}\limits_{i\in M,k\in N}\left[{A}_{\iota \kappa }\exp (-{B}_{\iota \kappa }{r}_{ij})-{C}_{\iota \kappa }{r}_{ik}^{-6}+{E}_{{{{{{\rm{elec}}}}}}}^{{{{{{{{\rm{DMA}}}}}}}}}({r}_{ik},{{{\Omega }}}_{ik})\right],$$

where *A*_ι*κ*_, *B*_ι*κ*_ and *C*_ι*κ*_ are empirically determined potential parameters describing the interactions between atoms of type ι and *κ*, *r*_*ik*_ is the distance between atoms *i* and *k* (in molecules *M* and *N*), of types *ι* and *κ*, and Ω_*ik*_ represents the relative orientation of the interacting atoms. Charge-charge, charge-dipole and dipole-dipole interactions were evaluated using Ewald summation. Higher order electrostatic and *exp-6* interactions were summed to a direct-space cut-off. The cut-off for both dispersion and electrostatic interactions was calculated as ${R}_{{{{{{\rm{cutoff}}}}}}}=\max$ (15.00 Å, *R*_intra_), where *R*_intra_ is the largest intramolecular atom-atom length, to ensure that the intermolecular interaction cutoff extends beyond the first shell of neighbours. To avoid the unphysical region of the *exp-6* interatomic potential, the distances between molecules were calculated after perturbation and the Monte Carlo move was rejected if any interatomic distance was shorter than the sum of covalent radii of the two elements +0.3 Å.

### Identification of connections between trajectories

The threshold algorithm does not involve local optimization of structures in principle. However, we require a method to identify whether a perturbation has led to a new energy basin. For this, perturbed structures are locally energy minimized if the perturbation is accepted (i.e., the unminimized energy was under the current lid energy). The minimized structures are compared to each other to identify where trajectories meet. Energy minimization is performed for every accepted perturbed structure because lattice energy landscapes are known to often contain many local minima around observed structures^12^, so every perturbation was assumed to potentially lead to a new local energy basin.

We found that this sometimes leads to huge unit cell with empty gaps between layers of molecules (see Supplementary Methods and Supplementary Fig. 19). To solve this issue, a three-step minimization process was applied as (1) initial optimization using FIT + DMA; (2) a second minimization with FIT + DMA with a small (0.1 GPa) pressure applied; (3) final reoptimization with FIT + DMA and no pressure. The first minimization without pressure is to ensure that the energy does not go uphill so that no energy barrier is overcome during local minimization. The second minimization with pressure applied is to compress the unit cell to remove artificial voids and the third step is to give the energy on the original energy landscape.

Two minima are considered to be connected at a given lid energy if the trajectories that start at or visit these minima are found to sample a common local minimum. Thus, the identification of identical structures (corresponding to the same local minimum) is an essential process to obtain the disconnectivity graph.

We use a two-step strategy: (1) a fast initial screen for duplicates is performed by comparison of simulated X-ray diffraction patterns, followed by (2) checking of duplicates using the COMPACK algorithm^56^, which compares interatomic distances and angles within a cluster of 30 molecules taken from the compared crystal structures (see full details in the Supplementary Methods).

Discussion of the convergence of threshold sampling is given in the Supplementary Methods (and Supplementary Figs. 20, 21).

### Structural descriptors and data featuring

Two descriptors of structural similarity—atom-centred symmetry functions (ACSFs)^46^ and the smooth overlap of atomic positions (SOAP)^45^—were used to investigate geometric clustering of crystal structures and their clustering into superbasins based on threshold simulations. Both approaches provide a measure of structural similarity based on comparison of local atomic environments.

ACSFs capture structural information from a series of radial and angular functions, which depend on neighbouring atomic positions out to a cutoff radius *R*_*c*_. We use ACSFs grouped by element, i.e. the functions are evaluated separately for all pairs (for radial functions) and triples (angular functions) of elements. The spacing and width of ACSFs is chosen as in the ANI-1 neural network force field^57^.

In the SOAP kernel, the local region of each atom is described individually by a sum of Gaussians centred on all atoms within the local environment. The approach applied here to calculate the similarity between two structures based on similarity of their atomic environments is the regularized-entropy match (REMatch) kernel.

Full details of ACSF and SOAP are provided in the Supplementary Methods.

Principal component analysis (PCA)^58^ and the clustering method HDBSCAN*^47^ are used to analyse the distribution of crystal structures in descriptor space, for comparison with the clustering into energy basins determined by the threshold simulations.

## Supplementary information

Day_PR File

Supplementary material

## Data Availability

Crystal structures generated by the threshold simulations, and details of their connectivity are available at 10.5258/SOTON/D2079.

## Code availability

The code used in this work is part of a wider code developed for crystal structure calculations and crystal structure prediction. This code is being prepared for release. Access can also be granted for non-commercial use by contacting Prof. Graeme Day.
