# Supplementary material for: Global analysis of the energy landscapes of molecular crystal structures by applying the threshold algorithm
Source: Commun Chem. 2022 Jul 28;5:86. doi: 10.1038/s42004-022-00705-4 (PMC9814927; doi:10.1038/s42004-022-00705-4)
Supplement: Supplementary file 2 — Supplementary material [file 42004_2022_705_MOESM2_ESM.pdf]

Supplementary Information for: Global analysis of the energy landscapes of molecular crystal structures by applying the threshold algorithm

Shiyue Yang and Graeme M. Day

School of Chemistry, University of Southampton, Southampton, SO17 1BJ, United Kingdom

1 Supplementary Notes

1.1 Threshold Results

CSP energy landscapes and disconnectivity graphs over the whole sampled energy range.

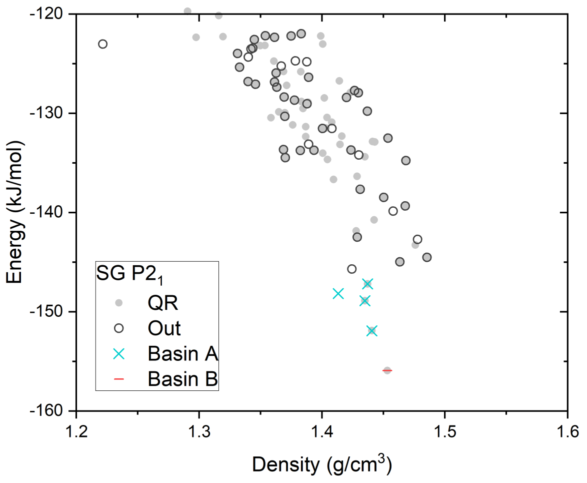

(a)

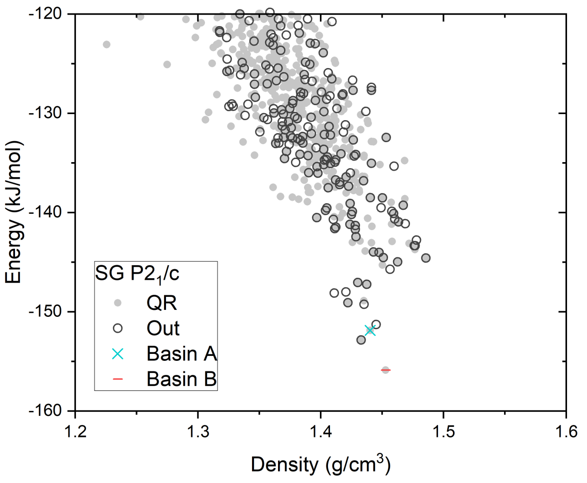

(c)

Supplementary Figure 1: Energy landscape compared between CSP using quasi-random sampling (QR) and from threshold simulations for indigo in space groups $P2_1$ and $P2_1/c$. Structures from threshold sampling are labelled as belonging to basin A, basin B or outside of both energy basins (OUT). The basins are defined as the disconnected sets of structures below the energy at which basins A and B are connected.

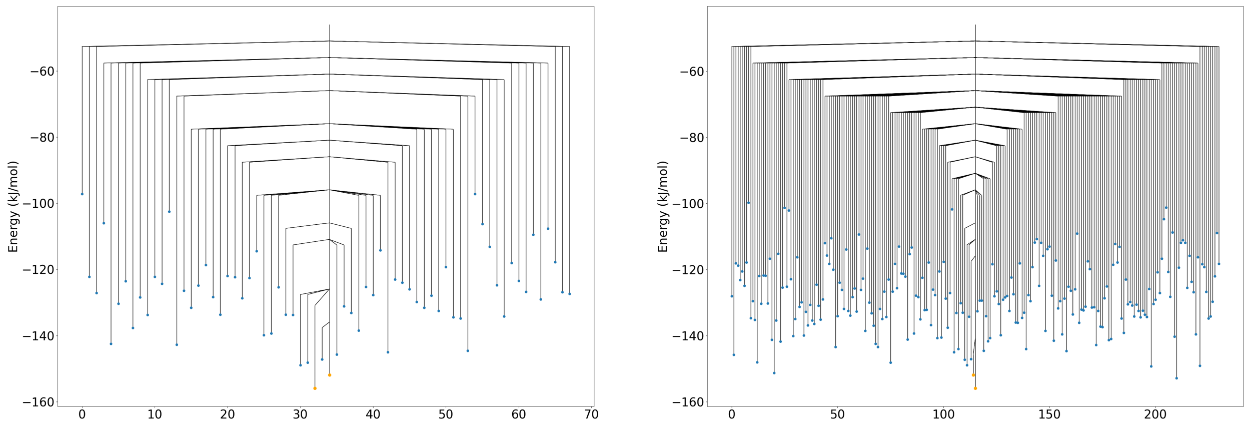

(a) (c)

Supplementary Figure 2: Connectivity graph from threshold simulations for indigo in space groups $P2_1$ and $P2_1/c$ over the whole sampled energy range.

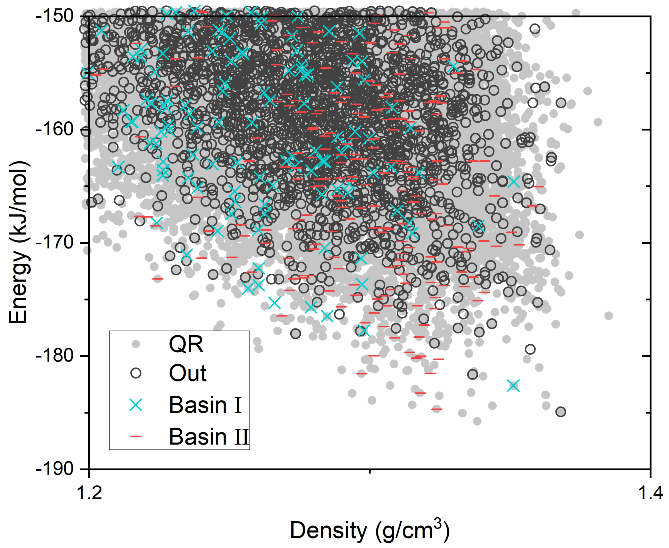

Supplementary Figure 3: Energy landscape compared between CSP using quasi-random sampling (QR) and from threshold simulations for the 1:1 nicotinamide:benzoic acid co-crystal in space group $P2_1/c$. Structures from threshold sampling are labelled as belonging to basin I, basin II or outside of both energy basins (OUT). The basins are defined as the disconnected sets of structures below the energy at which basins I and II are connected.

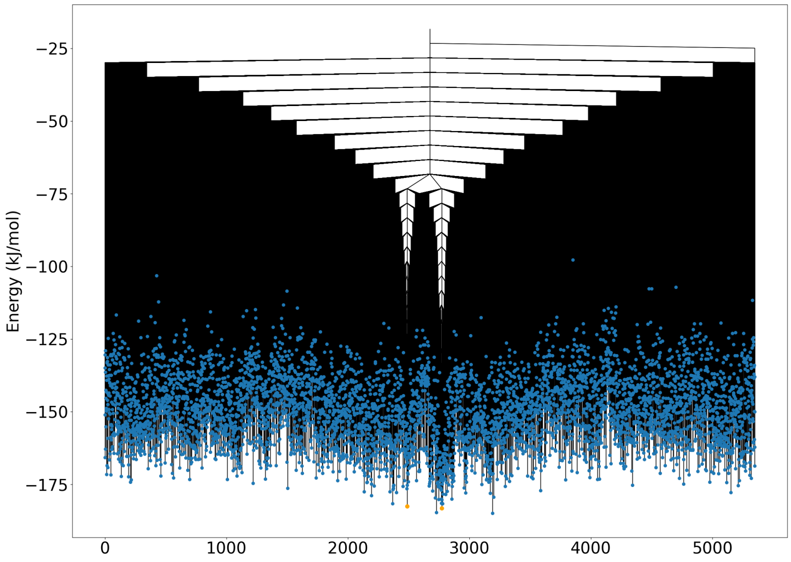

Supplementary Figure 4: Connectivity graph from threshold simulations for the 1:1 nicotinamide:benzoic acid co-crystal in space group and $P2_1/c$ over the whole sampled energy range.

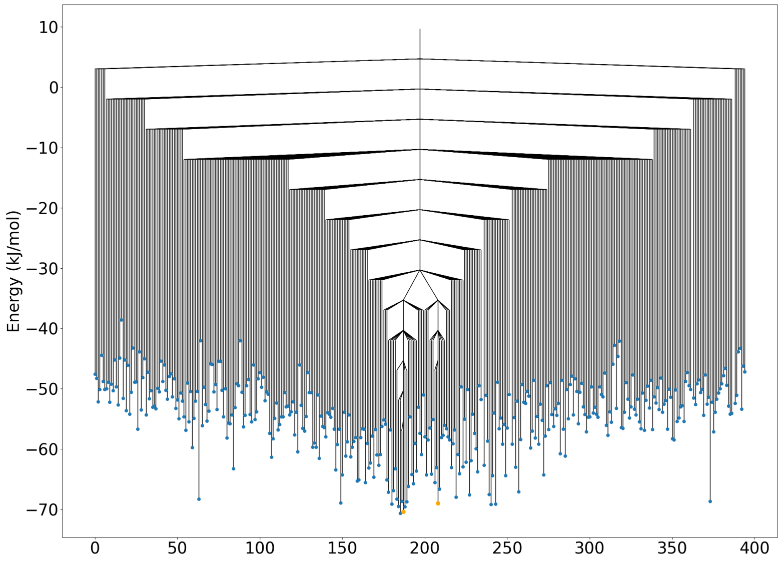

Supplementary Figure 5: Connectivity graph from threshold simulations for tetrolic acid in space group $P1$ over the whole sampled energy range.

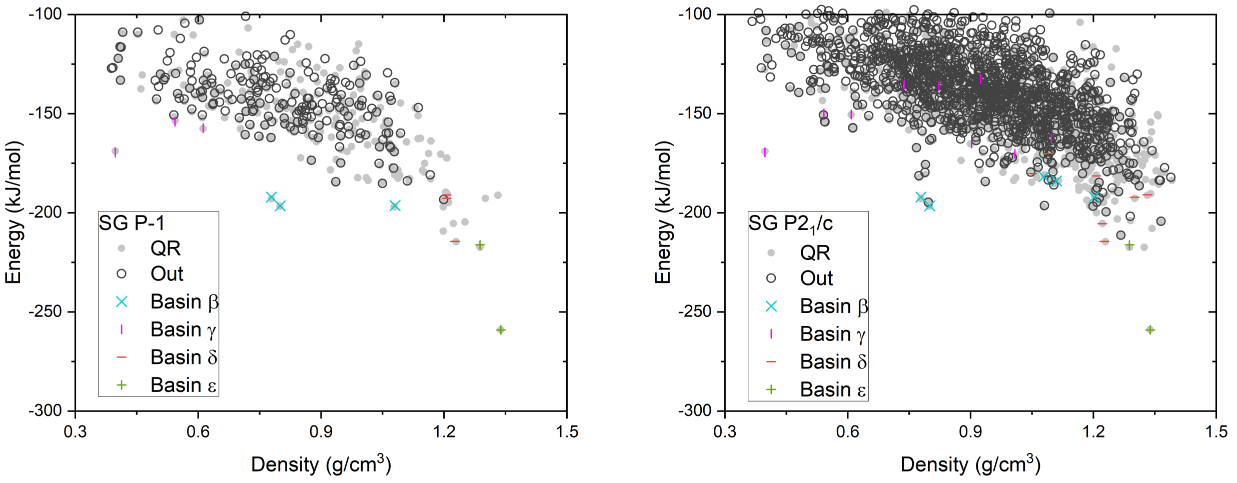

(a) (b)

Supplementary Figure 6: Energy landscape compared with quasi-random sampling from threshold simulations for TTBI in space group $P\bar{1}$ and $P2_1/c$. Structures from threshold sampling are labelled as belonging to basin β, γ, δ, ε or outside of both energy basins (OUT). The basins are defined as the disconnected sets of structures below the energy at which the basins are connected.

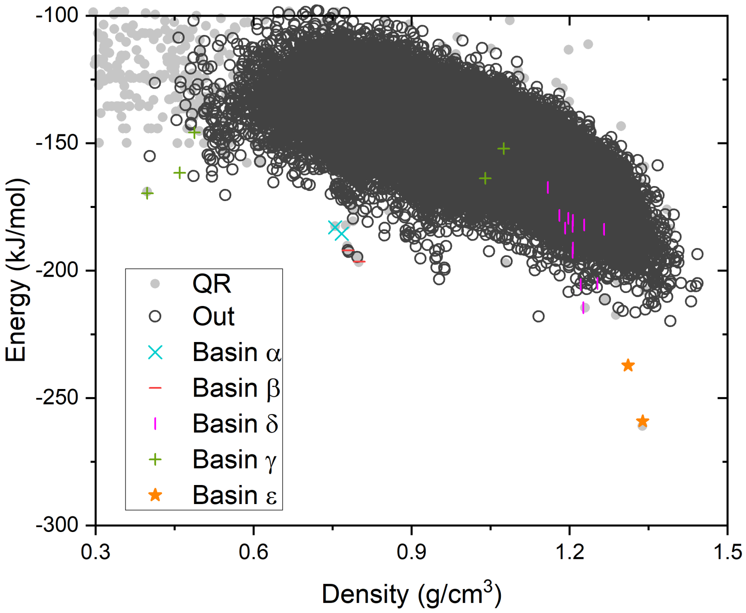

Supplementary Figure 7: Energy landscape compared with quasi-random sampling from threshold simulations for TTBI in with $Z = 4$ in a $P1$ unit cell. Structures from threshold sampling are labelled as belonging to basin α, β, γ, δ, ε or outside of both energy basins (OUT). The basins are defined as the disconnected sets of structures below the energy at which the basins are connected.

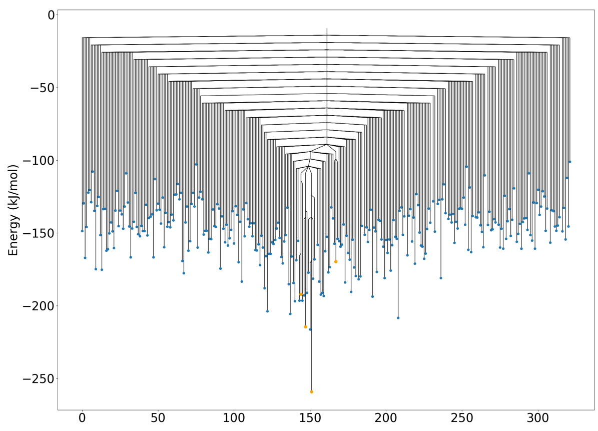

(a)

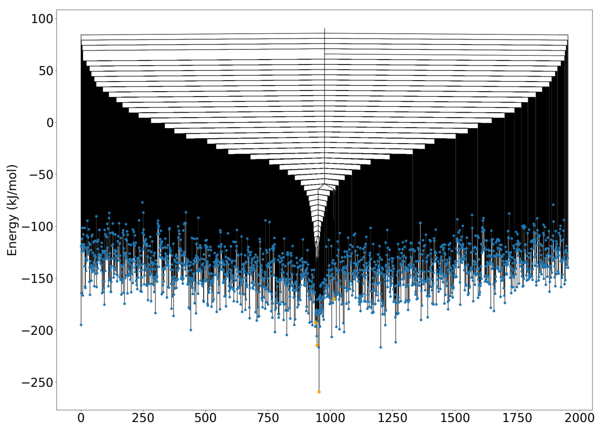

(c)

Supplementary Figure 8: Connectivity graph from threshold simulations for TTBI space groups $P\bar{1}$ and $P2_1/c$ over the whole sampled energy range.

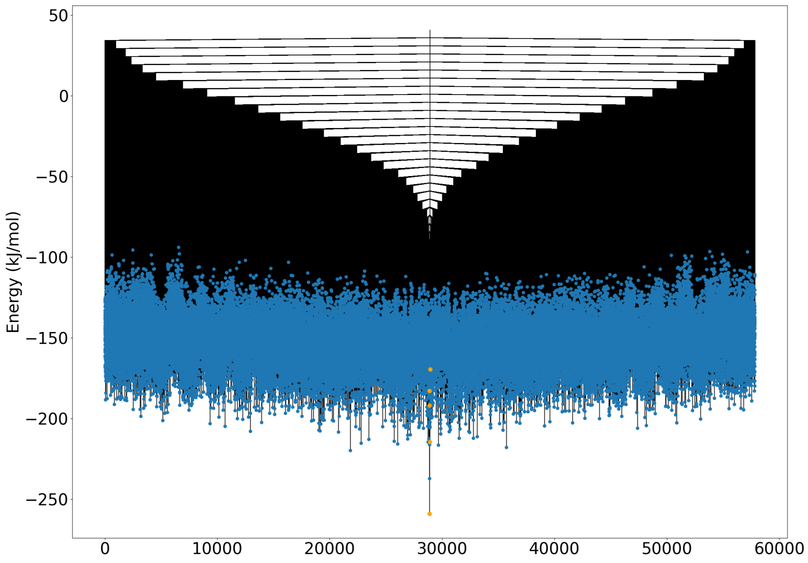

Supplementary Figure 9: Connectivity graph from threshold simulations for TTBI in $P1$ unit cell over the whole sampled energy range.

1.2 PCA and Clustering Results

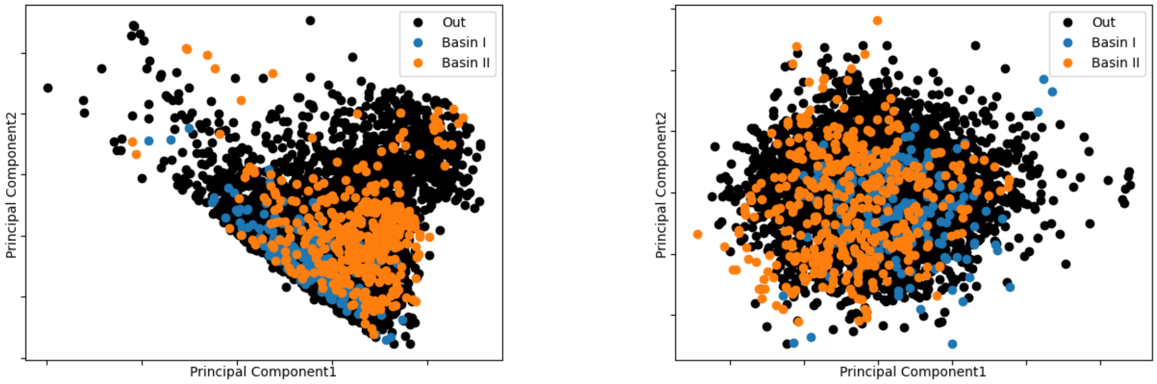

(a) (b)

Supplementary Figure 10: Projection of the structures sampled during the threshold simulations for the co-crystal GAZCES in space group $P2_1/c$, projected onto the first two principle components of (a) symmetry functions and (b) SOAP descriptors. Structures are colored by the energy basin that they occupy.

The HDBSCAN algorithm was applied for clustering with the parameter $min\_cluster\_size$, defining the minimum size of clusters, as 5, 10 or 20. HDBSCAN clusters are represented by the colour of edges on the disconnectivity graph, while non-clustered structures are represented with black edges.

For ACSFs, we tested three ways of calculating pairwise distances d between vector $\mathbf{r}^1$ and $\mathbf{r}^2$: the Euclidean distance

$$d(\mathbf{r}^1, \mathbf{r}^2) = \sqrt{(\mathbf{r}^1 - \mathbf{r}^2)^2} \tag{1}$$

the infinite order Minkowski distance:

$$d(\mathbf{r}^1, \mathbf{r}^2) = \max_i |\mathbf{r}_i^1 - \mathbf{r}_i^2| \tag{2}$$

where $\mathbf{r}_i$ is the ith component of $\mathbf{r}$, and by calculating the Euclidean distance between each atom and taking the maximal atomic distance as

$$d(\mathbf{r}^1, \mathbf{r}^2) = \max_{atom\ k} \sqrt{(\mathbf{r}_k^1 - \mathbf{r}_k^2)^2}. \tag{3}$$

Among these three distance metrics, infinite order Minkowski and maximal atomic approaches gave similar clustering results where almost all the structures were classified into one cluster (Supplementary Figure 13 and Supplementary Figure 14). Using Euclidean distances, neither pbserved structure was assigned to a cluster and all the structures in basin I were identified as noise (Supplementary Figure 12 (a)).

By varying the minimum cluster size, we found that either all the structures were identified as one huge cluster at $min\_cluster\_size = 5$ or the clusters vanished at larger size. Neither of two known crystal structures, **I** or **II**, are clustered into any group when $min\_cluster\_size$ is 10 or larger. The reason why the clusters vanishes with larger $min\_cluster\_size = 5$ is because the HDBSCAN requires the core structure to connect to sufficient neighbours to form clusters. In this case a lot of structures are only reachable to less than 10 other structures and thus identified as noise when $min\_cluster\_size$ exceeds 10. The failure to cluster the structures in individual basins indicates the dissimilarity between those structures.

The distribution of pairwise distances also indicated that there was no clear difference in distances between structures in the same basin and structures in different basins (Supplementary Figures 11 (d), 12(b), 13(b), 14(b)).

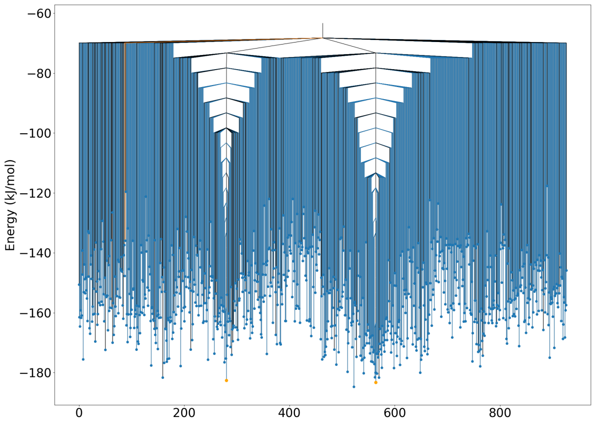

(a)

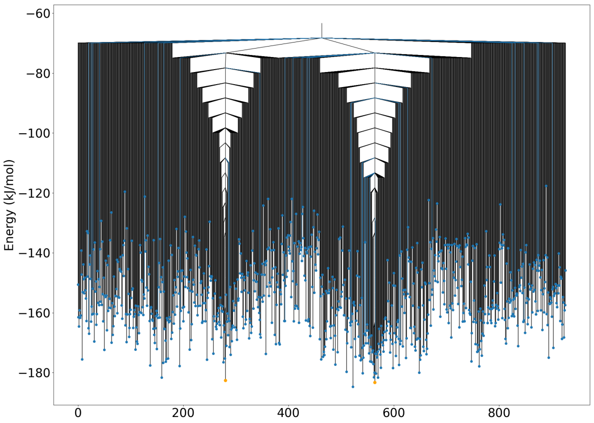

(b)

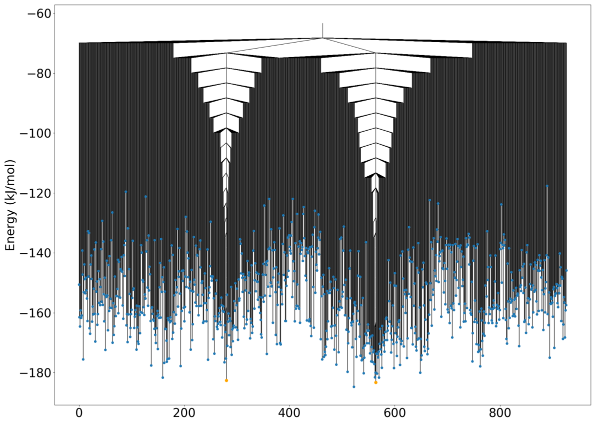

(c)

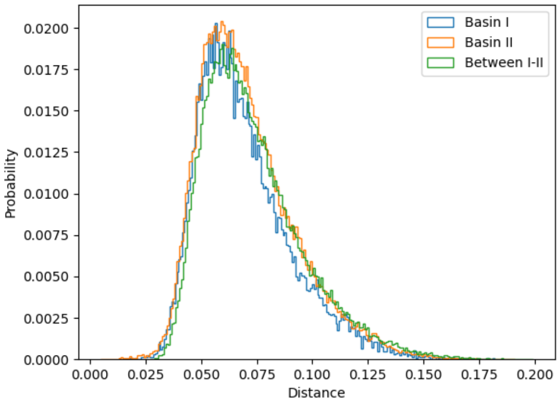

(d)

Supplementary Figure 11: Clustered connectivity graph for GAZCES $P2_1/c$ with SOAP REMatch kernel by HDBSCAN minimum cluster size at (a) 5, (b) 10 and (c) 20. Each color of edges implies a cluster while black ones represents noises. (b) Distribution of pairwise distance between structures within and between basins.

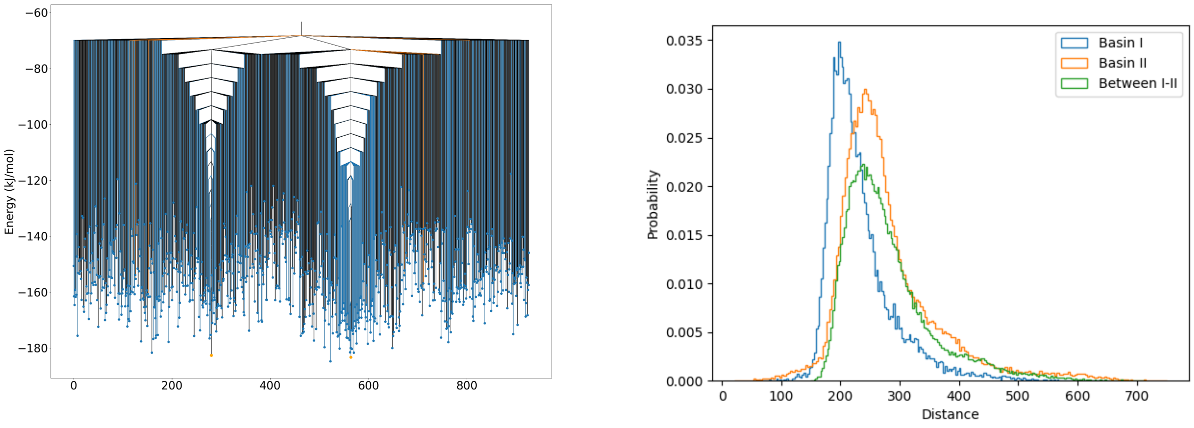

(a) (b)

Supplementary Figure 12: (a) Clustered connectivity graph for GAZCES $P2_1/c$ with symmetry function second order Euclidean distance. Each color of edges implies a cluster while black ones represents noises. (b) Distribution of pairwise distance between structures within and between basins.

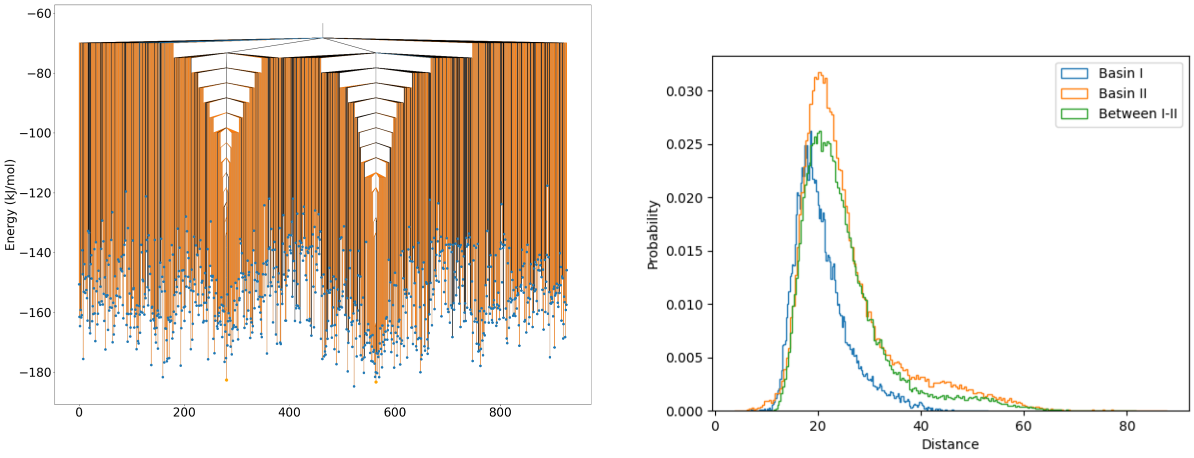

(a) (b)

Supplementary Figure 13: (a) Clustered connectivity graph for GAZCES $P2_1/c$ with symmetry function infinite order Minkowski distance. Each color of edges implies a cluster while black ones represents noises. (b) Distribution of pairwise distance between structures within and between basins.

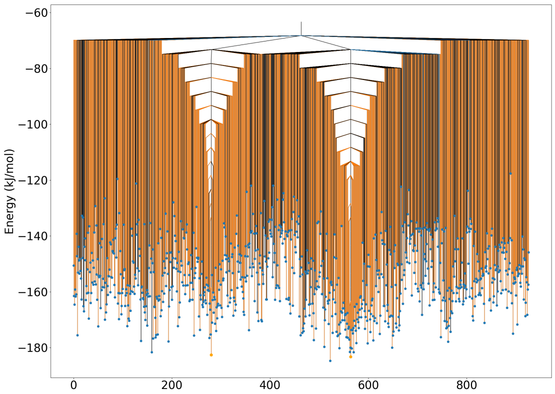

(a)

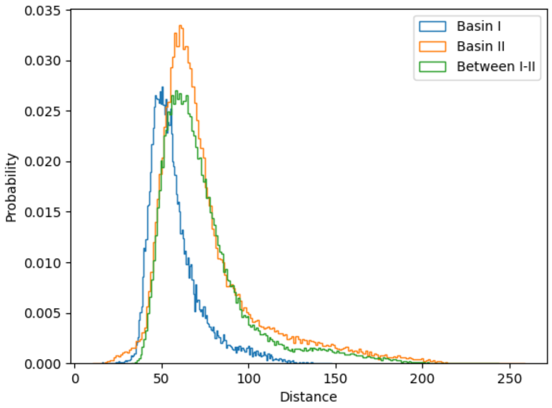

(b)

Supplementary Figure 14: (a) Clustered connectivity graph for GAZCES $P2_1/c$ with symmetry function maximal atomic distance. Each color of edges implies a cluster while black ones represents noises. (b) Distribution of pairwise distance between structures within and between basins.

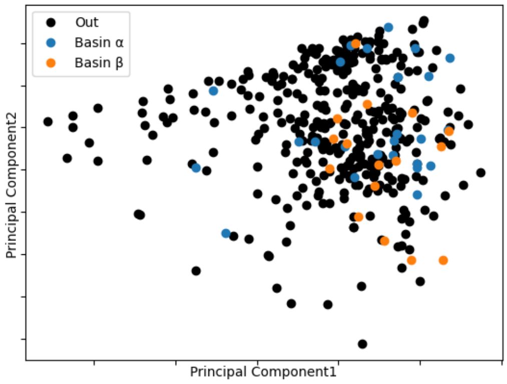

(a)

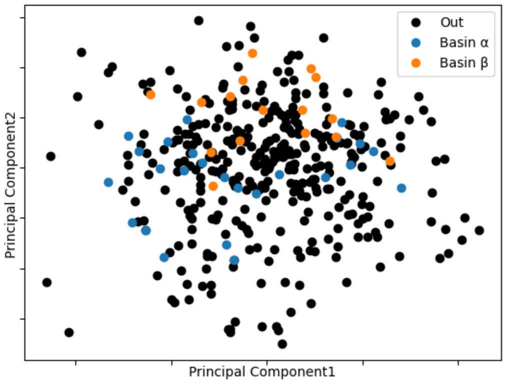

(b)

Supplementary Figure 15: Projection of the structures sampled during the threshold simulations for tetrolic acid in a $P1$ unit cell, projected onto the first two principle components of (a) symmetry functions and (b) SOAP descriptors. Structures are colored by the energy basin that they occupy.

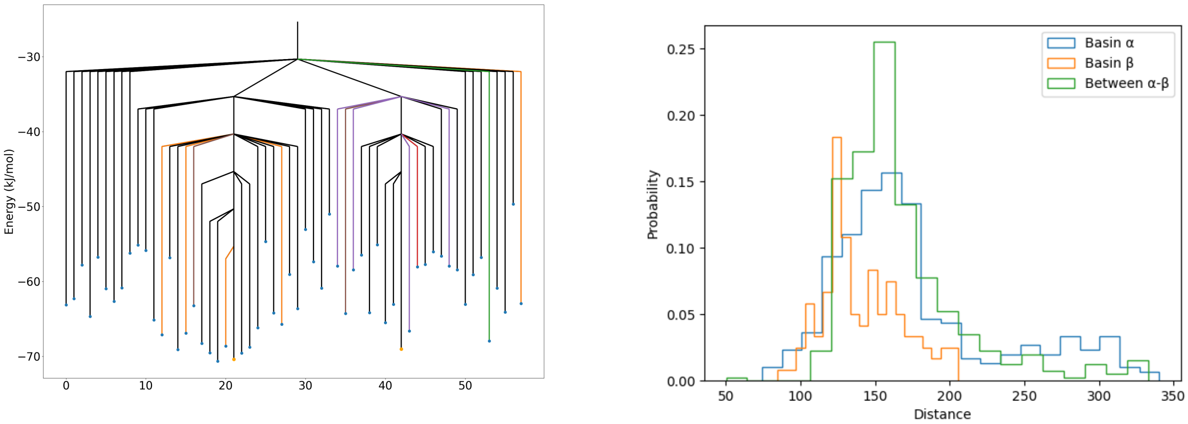

(a) (b)

Supplementary Figure 16: (a) Clustered connectivity graph for tetrolic acid in a $P1$ unit cell with symmetry function second order Euclidean distance. Each color of edges implies a cluster while black ones represents noises. (b) Distribution of pairwise distance between structures within and between basins.

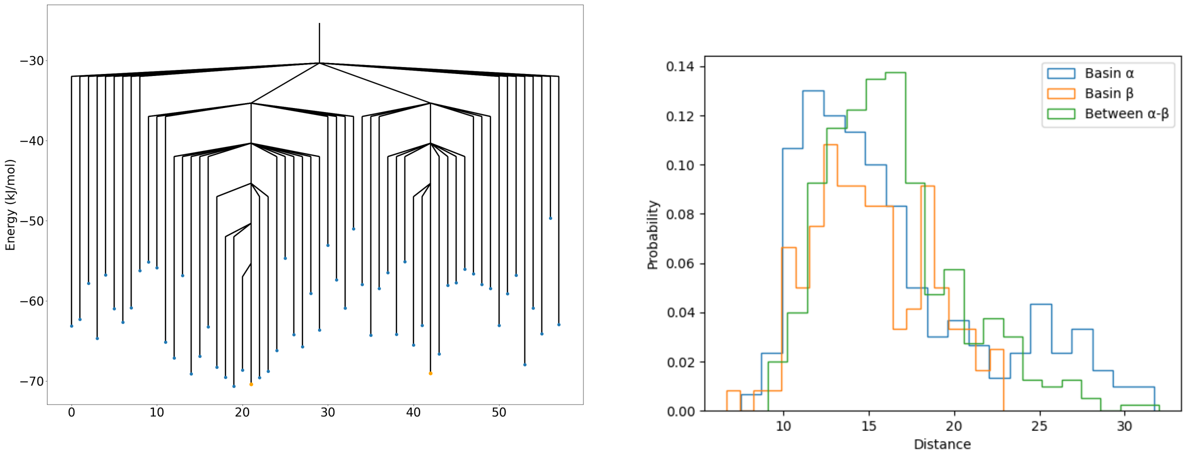

(a) (b)

Supplementary Figure 17: (a) Clustered connectivity graph for tetrolic acid in a $P1$ unit cell with symmetry function infinite order Minkowski distance. Each color of edges implies a cluster while black ones represents noises. (b) Distribution of pairwise distance between structures within and between basins.

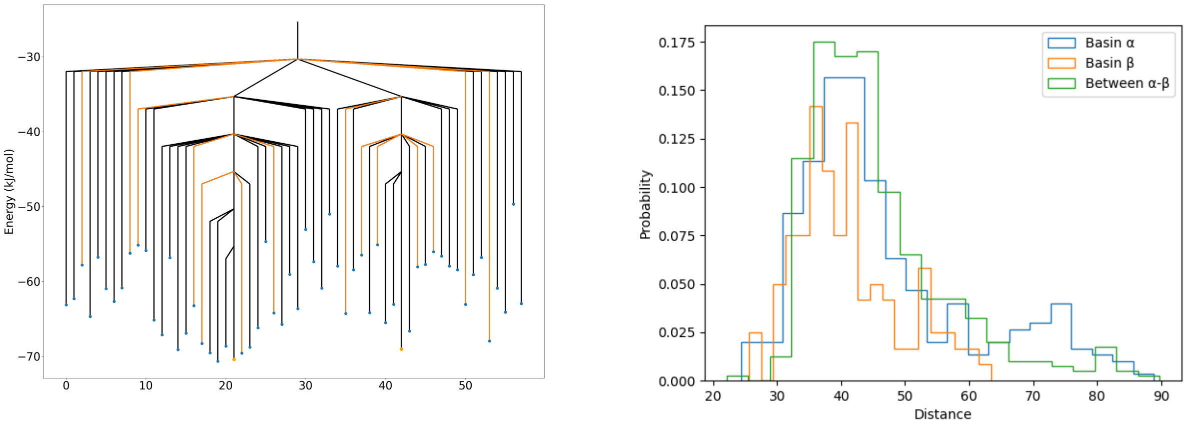

(a) (b)

Supplementary Figure 18: (a) Clustered connectivity graph for tetrolic acid in a $P1$ unit cell with symmetry function maximal atomic distance. Each color of edges implies a cluster while black ones represents noises. (b) Distribution of pairwise distance between structures within and between basins.

2 Supplementary Methods

2.1 Basin Identification

In the current strategy, the basin identification of a perturbed structure is by local energy minimization and duplicate comparison with database. In our first implementation of the threshold algorithm, in order not to include any possible uphill process to identify the correct minimum from a perturbed structure, the local energy minimization was a one-step approach: a direct minimization with FIT + DMA. However, we found that this strategy leads to some structures in which gaps have been introduced between layers of molecules. Taking the tetrolic acid threshold simulations in $P1$ as an example, we find sets of structures with the same energy (Supplementary Figure 19(a)), but with a wide density range. Consistently, the energy landscape over all density and energy indicates groups of structures at the same energy with density distributing from 0.3 to 0.75 g/cm^3, implying a loosely packed crystal structures. These structures are found to have layers of molecules divided by vacuum gaps (Supplementary Figure 19(b)). We find that these do not correspond to true energy minima, but occur because of the lack of significant intermolecular forces across these gaps. This is, in part, due to a cutoff that is applied to force field terms, which sets all interactions beyond the cutoff to zero to save computational expense. In our final implementation, and all results presented in this paper, these structures are removed by including an intermediate energy minimization step with a pressure applied, to remove such gaps. However, an alternative approach would be to leave these structures in and treat them as separate energy basins.

The key point here is what type of transitions should be allowed between crystal structures. In terms of energy landscape described by te energy potential with a distance cutoff, any structure should be included and thus those intermediates with vacuum gaps describe valid paths, although structures along the path do not correspond to equilibrium crystal structures. The transition between two crystal structures might go through states which are not crystals at all and these structures might suggest the need for volume expansion along the transition path. However it also makes sense if one is only interested in transition paths where the basins correspond to equilibrium crystal structures. In this case, a solution is to eliminate those structures with vacuum gaps without affecting those well packed structures. To ensure this, we apply the three-stage optimization. Most structures optimize to satisfactory crystal structures in the first step, and stay at the same energy minimum after application and removal of pressure in the second and third steps. However, those structures with vacuum gaps after the first step have these gaps removed when pressure is applied (to minimize the PV energy term) and are returned to the initial energy surface through re-optimization with the pressure term removed. One should bear in mind that here we are not focusing on local minimization, but the identification of which minimum the perturbed structure belongs to. Therefore both strategies are feasible and it is hard to say which one is better. One reason why we finally adopt the compressing minimization is because the duplicate identification of structures with vacuum gaps is quite difficult. The length of vacuum gap will not arouse energy variation with layer structure fixed, which forms a contour on the energy landscape. In principle, any structures on the contour should be identified as one unique structure to connect two structures with the contour in their transition path. However, due to lack of experience of dealing these vacuum gaps structures, we have not got an efficient way to identify these vacuum gap structures and cluster them. Therefore the compressed unit cells are finally utilized. If one wants to investigate all the transitions on the energy landscape, these contours formed by vacuum gaps should be taken into consideration and methods to deal with those structures need to be developed. In this project, we prefer all the intermediates still in crystal form and rule out all the minima with vacuum gaps.

2.2 Duplicate identification

Crystal structures were first compared using similarity of their calculated powder XRD patterns. Powder XRD patterns are calculated by Platon[1], normalized (such that their area under the curve was unity) and then compared with each other by the constrained Dynamic Time Warping (CDTW) algorithm, which is the summation of Euclidean distance with Sakoe-Chiba global constraint and window length being 10. The tolerances for comparing pairs of structures were 1.0 kJ·mol^{-1} and 0.05 g·cm^{-3}.

Matched structures were checked using the COMPACK algorithm,[2] which compares the intermolecular environment between two crystal structures. As to the intermolecular environment comparison, a 30-molecule cluster is constructed surrounding the molecules in the asymmetric unit for the reference and comparison structures. The origins of two clusters are positioned at the same point and the best overlapping for every molecule is searched by the action of rotation only[3]. The two molecules are considered matched if interatomic distances are within 20% tolerance and angles are within 20 degrees tolerance. The current tolerance for an identical pair is a match of 30 out 30 molecules with root mean squared distance less than 0.5 Å.

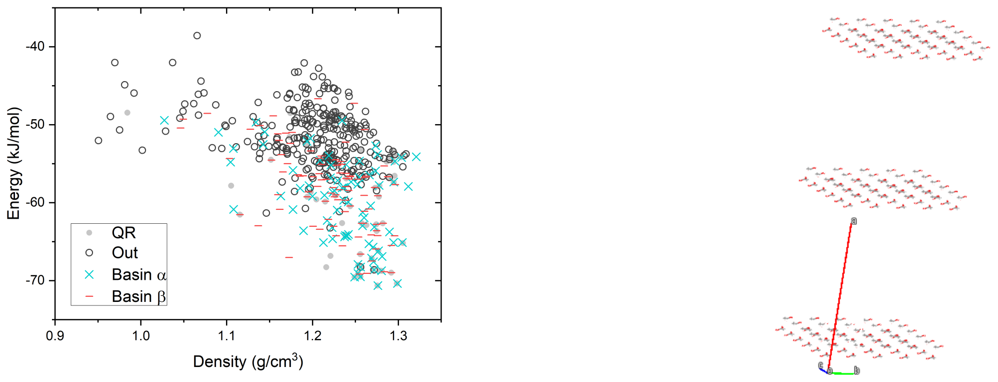

(a) (b)

Supplementary Figure 19: (a) Landscape compared with quasi-random sampling over all the density and energy range for tetrolic acid in $P1$ uni cell. (b) An example of structure with vacuum gap between layers of structures.

2.3 Convergence and adaptive sampling

The threshold algorithm trajectories were run for fixed numbers of steps. We also implemented methods with flexible number of steps automatically adjusted under each lid energy, but have not found a satisfactory convergence criterion, so suggest the fixed step method for now.

Several criteria were applied and tested, using the benzamide crystal structure landscape in space group $F2dd$ as a test. The idea was that more steps are needed as the lid energy increases, due to a larger accessible configurational space. The first property tested for monitoring convergence was the acceptance ratio. Three simulations were run under three lid energies: 5 kJ/mol, 25 kJ/mol and 50kJ/mol from the same initial structure. The variation of acceptance ratio with iteration is shown in Supplementary Figure 20 (a) for each lid energy. The acceptance ratio increased with the rising lid energy as our expectation. The variance of the acceptance ratio converged to 0 as the trajectories proceeded, but the rate of convergence was not what we expected (Supplementary Figure 20 (b)). The rate of convergence were similar under 25 kJ/mol and 50 kJ/mol and thus could not distinguish the length of simulations reqired for these two lid energies.

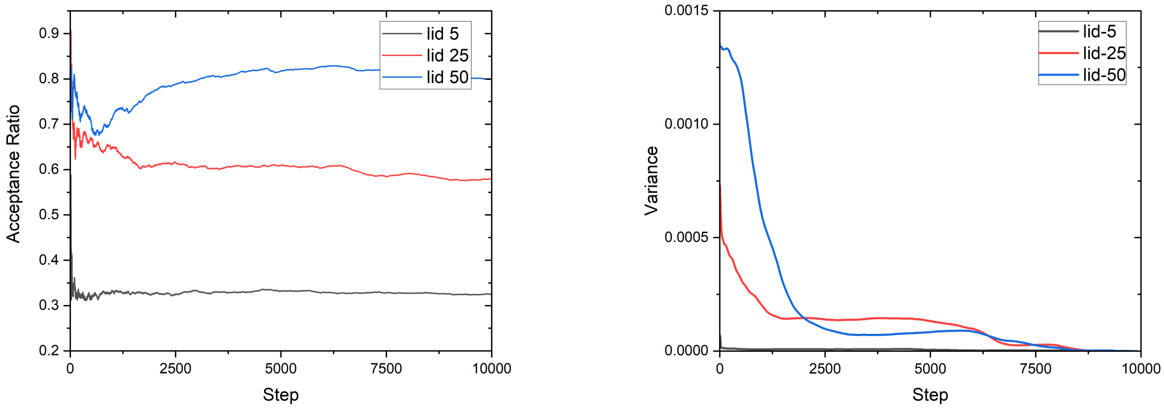

(a) (b)

Supplementary Figure 20: (a) Acceptance ratio of threshold Monte Carlo steps under three lid energies for benzamide in space group $F2dd$. (b) Variance of the acceptance ratio for simulations with three lid energies.

The second property investigated was the ratio of structures with energy within 1 kJ/mol window from lid energy. This could be viewed as a projection of energy distribution (Supplementary Figure 21 (a)). However, the convergence rate of this ratio was not found to vary systematically with lid energy (Supplementary Figure 21 (b)): the ratio converged fastest at a lid energy of 25 kJ/mol.

Identification of suitable metrics for monitoring convergence of sampling is, therefore, left for future development.

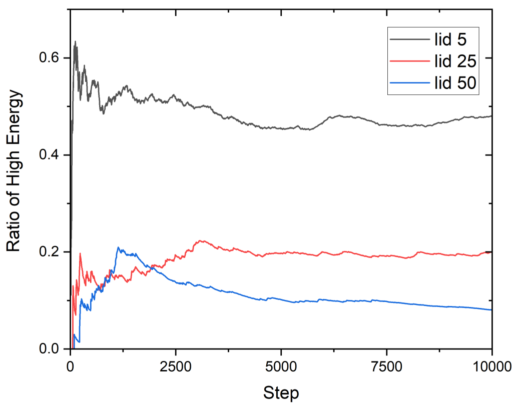

(a)

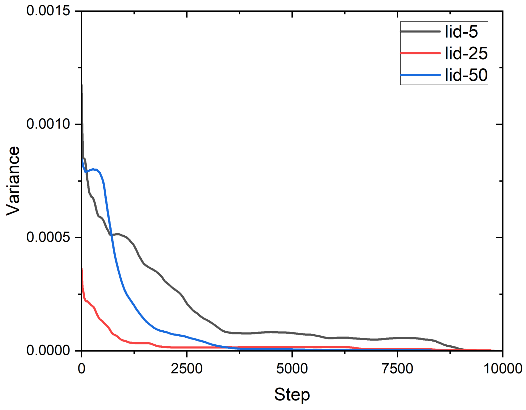

(b)

Supplementary Figure 21: (a) The ratio of high energy structures (defined as those within 1 kJ/mol of the lid energy) from threshold Monte Carlo steps under three lid energies for benzamide in space group *F*2*dd*. (b) Variance of the ratio of high energy structures with three lid energies.

2.4 Descriptors

2.4.1 Atom-centered symmetry functions

Atom-centered symmetry functions (ACSFs) are a descriptor of local atomic environments that are invariant to translation, rotation and permutation[4]. The symmetry function describes an N-atom system by an atomic environment vector $\mathbf{G} = \{G_1, G_2, ..., G_N\}$ where G_i represents the ith atom's radial and angular chemical environment individually implying two-body and three-body interactions. In the actual numerical calculation, G_i approximates both radial and angular features in a local environment and the local environment is defined by a piece-wise cutoff functions

$$f_c(R_{ij}) = \begin{cases} \frac{1}{2}\cos\left(\frac{R_{ij}}{R_c}\pi\right) + \frac{1}{2}, & R_{ij} \leq R_c \\ 0, & R_{ij} > R_c. \end{cases} \quad \text{(Supplementary Equation (1))}$$

where R_c is the cutoff radius. The cutoff function f_c is designed to be continuous and differentiable, and ensures that the symmetry functions tend to 0 with distance exceeding the cutoff radius.

The radial functions are constructed as sums of two-body terms as

$$G_m^R = \sum_{j\neq i}^{N} \exp\left[-\eta(R_{ij} - R_m^s)^2\right] f_c(R_{ij}) \quad \text{(Supplementary Equation (2))}$$

where η and R_s individually represents the width and center of Gaussian, and index m indicates a specific set of R^s parameters. In this work multiple R^s are used to probe specific regions of radial environments. With the same width, the center of Gaussian shifting with R^s and height of the radial function is controlled by the cutoff function.

The angular functions are constructed as summations of three-body cosine functions of the angles $\theta_{ijk} = \arccos(\mathbf{R}_{ij} \cdot \mathbf{R}_{ik}/|R_{ij}R_{ik}|)$ as

$$\begin{aligned} G_m^A =& 2^{1-\zeta} \sum_{j,k\neq i} [1 + \cos(\theta_{ijk} - \theta_m^s)]^\zeta \\ &\times \exp\left[-\eta\left(\frac{R_{ij} + R_{ik}}{2} - R_m^s\right)^2\right] f_c(R_{ij}) f_c(R_{ik}). \end{aligned} \quad \text{(Supplementary Equation (3))}$$

The parameter θ^s shifts the local angular environment, while R^s here controls the radial centre of the function. The two parameters controlling width of distributions η and ζ are kept constant throughout calculations.

With the above approaches, for each atom a radial and angular vector is obtained by caluclation of the symmetry functions at a series of parameters. However,in its original form, the local environment is only different on atomic positions and not distinguishable with elements, i.e., atomic numbers, which would find difficulty identifying different atoms at similar distance. To solve this issue, rather than summing over all elements, the local environments are represented by a radial part for each atomic number and a angular function for each atomic pair, which gives L radial vectors and $L(L-1)/2$ angular vectors for L atomic numbers[5]. The dimension of a atomic environment vectors are larger than the degrees of freedom of the system, ensuring enough dimensionality and thus features of the system captured. The symmetry functions are able to describe symmetrical features with identification of elements and perform better in diverse chemical environments.

2.4.2 SOAP REMatch Kernel

In the SOAP kernel, the local region of each system is described individually, expanded by a set of orthonormal basis functions and a similarity matrix between systems is evaluated by dot products to simplify the calculation. The matrix containing the complete information on the pairwise similarity is further transformed by various approaches, in our case, REMatch kernel.

The basic idea of SOAP descriptor is comparing structures based on distributions of atoms in the local environment of each atom. The local density of atoms is represented by a sum of Gaussians centering on all the atoms within the local environment χ as

$$\rho_\chi(\mathbf{r}) = \sum_{i\in\chi} \exp\left[-\frac{(\mathbf{x}_i - \mathbf{r})^2}{2\sigma^2}\right] \quad \text{(Supplementary Equation (4))}$$

where σ is the variance of the Gaussian. In a similar way to symmetry functions, a cutoff function is multiplied by the atomic density to select the atoms from central atom smoothly. The SOAP kernel defines the overlap $\tilde{k}$ between two environments χ and χ' by the integral over the space as well as rotations $\hat{R}$ in three dimensions

$$\tilde{k}(\chi, \chi') = \int \mathrm{d}\hat{R} \left| \int \mathrm{d}\mathbf{r} \rho_{\chi}(\mathbf{r}) \rho_{\chi'}(\hat{R}\mathbf{r}) \right|^2 . \quad \text{(Supplementary Equation (5))}$$

The obtained kernel is usually normalized so that the self-similarity is unity as

$$k(\chi, \chi') = \frac{\tilde{k}(\chi, \chi')}{\sqrt{\tilde{k}(\chi, \chi) \cdot \tilde{k}(\chi', \chi')}}. \quad \text{(Supplementary Equation (6))}$$

With the integral over rotation operators, the SOAP kernel is invariant to translation, rotation and permutation. In actual calculation, the normalized SOAP kernel is given as[6]

$$k(\chi, \chi') = \hat{\mathbf{p}}(\chi) \cdot \hat{\mathbf{p}}(\chi'). \quad \text{(Supplementary Equation (7))}$$

where $\mathbf{p}(\chi)$ is the normalized power spectrum vector. Therefore, given two structures with N atoms, a N^2 local similarity matrix is obtained by the calculating pairwise similarity kernel, or distance given as

$$d(\chi, \chi') = \sqrt{2 - 2\hat{\mathbf{p}}(\chi) \cdot \hat{\mathbf{p}}(\chi')}. \quad \text{(Supplementary Equation (8))}$$

which will always hold since $\mathbf{p}$ is a unit vector.

Given a database of structures as input, the power spectrum vector $\mathbf{p}$ is calculated individually for each structure, and the local similarity matrix is estimated for each pair of structures. Then the question is how to evaluate the similarity of two systems with the local similarity matrix. Here we note matrix $\mathbf{C}(A, B) = k(\chi^A, \chi^B)$ and component $C_{ij}(A, B) = k(\chi_i^A, \chi_j^B)$ for structures A and B. The approach applied here is to calculate the similarity $K(A, B)$ is the regularized-entropy match (REMatch) kernel, defined as

$$\begin{aligned} &\hat{K}^{\gamma}(A, B) = \mathrm{Tr}\mathbf{P}^{\gamma}\mathbf{C}(A, B), \\ &\mathbf{P}^{\gamma} = \underset{\mathbf{P} \in \mathcal{U}(N,N)}{\arg\min} \sum_{ij} P_{ij}(1 - C_{ij} + \gamma \ln P_{ij}). \end{aligned} \quad \text{(Supplementary Equation (9))}$$

Parameter γ controls the contribution of entropy penalty and the calculated similarity is normalized to make self-similarity unity.

References

[1] A. Spek, "Platon/squeeze," *Acta Crystallogr., Sect. D: Biol. Crystallogr*, vol. 65, pp. 148–155, 2009.

[2] J. A. Chisholm and S. Motherwell, "Compack: a program for identifying crystal structure similarity using distances," *Journal of applied crystallography*, vol. 38, no. 1, pp. 228–231, 2005.

[3] E. A. Coutsias, C. Seok, and K. A. Dill, "Using quaternions to calculate rmsd," *Journal of computational chemistry*, vol. 25, no. 15, pp. 1849–1857, 2004.

[4] J. Behler, "Atom-centered symmetry functions for constructing high-dimensional neural network potentials," *The Journal of chemical physics*, vol. 134, no. 7, p. 074106, 2011.

[5] J. S. Smith, O. Isayev, and A. E. Roitberg, "Ani-1: an extensible neural network potential with dft accuracy at force field computational cost," *Chemical science*, vol. 8, no. 4, pp. 3192–3203, 2017.

[6] A. P. Bartók, R. Kondor, and G. Csányi, "On representing chemical environments," *Physical Review B*, vol. 87, no. 18, p. 184115, 2013.
