# Supplementary material for: Global analysis of the energy landscapes of molecular crystal structures by applying the threshold algorithm
Source: Commun Chem. 2022 Jul 28;5:86. doi: 10.1038/s42004-022-00705-4 (PMC9814927; doi:10.1038/s42004-022-00705-4)
Supplement: Supplementary file 1 — Day_PR File [file 42004_2022_705_MOESM1_ESM.pdf]

nature portfolio

Peer Review File

Global analysis of the energy landscapes of molecular crystal structures by applying the threshold algorithm

Open Access This file is licensed under a Creative Commons Attribution 4.0 International License, which permits use, sharing, adaptation, distribution and reproduction in any medium or format, as long as you give appropriate credit to the original author(s) and the source, provide a link to the Creative Commons license, and indicate if changes were made. In the cases where the authors are anonymous, such as is the case for the reports of anonymous peer reviewers, author attribution should be to 'Anonymous Referee' followed by a clear attribution to the source work. The images or other third party material in this file are included in the article's Creative Commons license, unless indicated otherwise in a credit line to the material. If material is not included in the article's Creative Commons license and your intended use is not permitted by statutory regulation or exceeds the permitted use, you will need to obtain permission directly from the copyright holder. To view a copy of this license, visit http://creativecommons.org/licenses/by/4.0/.

Reviewers' comments:

Reviewer #1 (Remarks to the Author):

This paper investigates the method of Monte-Carlo threshold algorithm as a means of producing semi-quantitative connectivity graphs to show the barrier to interconversion between crystal structures of the same rigid organic molecule. The aim is to detect which crystal structures have such a low barrier to interconversion that only the lowest could be observed. The paper will be of immense interest to those interested in the crystallisation of organic molecules, particularly pharmaceuticals, as a possible solution to the over-prediction problem of crystal structure prediction. This is a severe problem, as current CSP methods produce many more minima in the lattice energy (static structures approximating a temperature of 0 K) than observed polymorphs. Structures which are connected by a very high barrier could result in considerable problems in quality control of the crystallized product, as crystallization may produce a kinetically-favored metastable polymorph consistently until the first nucleation of a thermodynamically more stable form. Other polymorphic systems have low barrier such that the polymorphs can transform to each other depending on conditions of temperature and pressure. This method seeks to determine when the barrier is so low, that only one of the structures can be ever observed experimentally. The aim is important, and the paper innovates a reasonable methodology that has only previously been applied to simple inorganic systems. The difficulty that this paper clearly illustrates is that of the periodic nature of the crystal and choosing a large enough unit cell that it can represent sufficient structures and yet be computationally tractable. As very few phase transitions between organic crystals occur maintaining translational symmetry (~ martensitic/second order/reversible single-crystal to single-crystal), computational modelling that enforces this order is not a realistic model of phase transitions. The authors clearly demonstrate that the barriers predicted are very dependent on cell size, even to within the 5 kJ/mol use as the level of discrimination as the increments in step size. Furthermore, the basins visited starting from a particular structure, either the global minimum in the CSP or the experimental structures does not cover all the structures found in the CSP within that space-group constraint. The paper clearly illustrates the key considerations in using this approach to reduce the entire CSP landscape. It then concentrates on what the method can reveal about the known polymorphs of the four systems. This is very effective and revealing, particularly for the surprising stability of the low density, highly metastable polymorphs of TTBI. This is clearly an effective method of showing the relationship between known polymorphs and understanding their properties in terms of the energy barriers.
It is a bit surprising that the attempts to show how similar the connected structures are using two similarity measures, atom-centered symmetry functions (ACSFs) and the smooth overlap of atomic positions (SOAP) seems inconclusive. However, this may only reflect the nature of the 1:1 nicotinamide:benzoic acid co-crystal disconnectivity tree structure meaning that the structures are so different. Does the one example justify the third paragraph in the conclusion (p 11 l720) being there rather than in the discussion? The issue as to whether these methods could automate human judgement of structures whether structures are sufficiently closely related that only the most stable is likely to result from any crystallization process, or the structures may appear as disorder/stacking defects the structure, has to be system-dependent. Interpreting the results of this study also requires insight into the extent of experimental polymorph screening.

The cost of the calculations is not commented upon. It will not be cheap, as at each accepted MC step a multi-stage minimisation is performed to establish whether the structure has gone over the barrier to another structure. This requires the potential energy surface to be described relatively

cheaply, but this MC based method is capable of using more accurate atomistic modelling than the recently proposed method of reducing the crystal energy landscape by doing Molecular Dynamics simulations. It is also a requirement that could be speeded up in development of the methodology. The choice of systems is good, within the constraint that the molecules have to be rigid and illustrate the differences between polymorphs with the same or different hydrogen bonding, same or different space-groups. This is pioneering work that is described in sufficient technical detail that the concepts and considerations are clear, so that others could decide whether it would be worth developing this type of approach for their specific system and application.
This paper will influence thinking about how the observation of polymorphs is related to the nature of the potential energy wells, rather than just their depth. It is a challenge to the current practice of using energy cutoffs in CSP, a practice that does not account for the observation of long-lived highly metastable pharmaceutical polymorphs when solvent removal is very late in the crystallization process (e.g. desolvated solvates). It also shows that there is no quick fix for the problem of reducing the over-prediction problem of lattice energy based organic crystal structure prediction that arises from the neglect of the molecular motion arising from temperature. It clearly shows the conceptual and technological demands of modelling crystals and their transitions at finite temperature. I strongly recommend publication, after the authors have considered the following points:
Please label the horizontal axis in the disconnectivity graphs, explaining the difference in Fig 4, so that it is clear for the other examples.
Comparing Table 1 and 2 is difficult on screen. Given that the information can be estimated from Fig 11, consider polishing the TTBI results to be one discussion of the relationship between the polymorphs, and another on the problem of choosing the simulation cell for the algorithm.
The three stage optimization requirement is well explained in S3.1, which reflects the key point, that some transitions can only go via an unphysical mechanism, such as large separation between layers, because of the imposition of translational symmetry. This is a point worth a few sentences in the discussion.

Reviewer #2 (Remarks to the Author):

In this manuscript, the authors present a numerical implementation of the Monte Carlo threshold algorithm for estimating the energy barriers between different polymorphs. They also applied their tool to investigate for different cases with and without space group symmetry constraint. Their results suggests that their tool can adequately sample the energy surface and generate an easily interpretable connectivity map between the local energy minima. An immediate application of this method would be to check if the metastable polymorph can be kinetically stable.

I have only two minor suggestions.
1. It is said the implementation is very similar to basin hopping. Can you give the exact procedure for each MC perturbation. For instance, which variables were perturbed by how many magnitude?. Such details will be useful for the readers to gain a better understanding.

2, The authors briefly mentioned their work was inspired from some previous studies on the inorganic systems like MgF_2 and CaF_2. In fact, there have been some similar attempts for estimating the metastability for the newly predicted inorganic compounds (e.g Zhu and Oganov, CrystEngComm 14 (10), 2012). Such work should be mentioned.

Overall, this is a very high quality work in my opinion. It should be published without any doubts.

Reviewer #3 (Remarks to the Author):

The work by Shiyue Yang and Graeme Day presents a very interesting approach to identify relevant crystal restructures in crystal energy landscapes by estimating the energy barriers separating the experimental and predicted crystal structures.
From the scientific contribution, the presented approach can be seen as a guideline how to interpret crystal energy landscapes and may be accepted for publication after following points have been addressed:
1. A lot of known information, i.e. the experimental structures, went into the design of the work. In reality, especially for pharmaceutical and other fine-chemical industries, one of the goals of a CSP study is to identify other relevant polymorphs. The authors used the experimental structures and low(est) energy structures as staring points for the threshold simulations. How will the results be affected if other structures are used as staring points? Which structures should be chosen in case the experimental structure(s) has/have not been determined?
2. The chosen examples are all based on rigid-body molecules. How will flexibility of the molecules affect this method and the cut-offs that have to be chosen?
3. How expensive (computationally) is the additional step compared to the generation of the crystal energy landscapes?
4. 2.3. Identification of connections between trajectories - concerning the cut-off that was applied to define whether a perturbation that has led to a new energy minimum. How big is the risk that a structure is discharged in case the perturbated unminimized geometry is too far from the minimized geometry/structure? Isn't the applied method at risk of losing structures (basins)?
5. 3.1. Sampling within a space group. Even in case two polymorphs adopt the same space group symmetries transformations/connections between those might still be able at a lower energy cost via lower symmetry relations. Was this approach considered in the two chosen examples?
6. 3.2.2. TTBI example: The lattice energy landscape suggests that the most stable polymorph has not been found yet. Is the big energy gap an artifact of the chosen lattice energy minimization method? The lattice energy difference of approx. 50 kJ/mol seems to be high, especially for a fairly rigid molecule and the fact that higher energy structures fall into the same density range.
7. 3.2.2. TTBI example, last paragraph. The statement "The result is in line with the experimentally observed transformation between the a and b polymorph." has to be elaborated. How was the value determined (in literature)?

Reviewer #1 (Remarks to the Author):

This paper investigates the method of Monte-Carlo threshold algorithm as a means of producing semi-quantitative connectivity graphs to show the barrier to interconversion between crystal structures of the same rigid organic molecule. The aim is to detect which crystal structures have such a low barrier to interconversion that only the lowest could be observed. The paper will be of immense interest to those interested in the crystallisation of organic molecules, particularly pharmaceuticals, as a possible solution to the over-prediction problem of crystal structure prediction. This is a severe problem, as current CSP methods produce many more minima in the lattice energy (static structures approximating a temperature of 0 K) than observed polymorphs. Structures which are connected by a very high barrier could result in considerable problems in quality control of the crystallized product, as crystallization may produce a kinetically-favored metastable polymorph consistently until the first nucleation of a thermodynamically more stable form. Other polymorphic systems have low barrier such that the polymorphs can transform to each other depending on conditions of temperature and pressure. This method seeks to determine when the barrier is so low, that only one of the structures can be ever observed experimentally. The aim is important, and the paper innovates a reasonable methodology that has only previously been applied to simple inorganic systems. The difficulty that this paper clearly illustrates is that of the periodic nature of the crystal and choosing a large enough unit cell that it can represent sufficient structures and yet be computationally tractable. As very few phase transitions between organic crystals occur maintaining translational symmetry (~ martensitic/second order/reversible single-crystal to single-crystal), computational modelling that enforces this order is not a realistic model of phase transitions. The authors clearly demonstrate that the barriers predicted are very dependent on cell size, even to within the 5 kJ/mol use as the level of discrimination as the increments in step size. Furthermore, the basins visited starting from a particular structure, either the global minimum in the CSP or the experimental structures does not cover all the structures found in the CSP within that space-group constraint. The paper clearly illustrates the key considerations in using this approach to reduce the entire CSP landscape. It then concentrates on what the method can reveal about the known polymorphs of the four systems. This is very effective and revealing, particularly for the surprising stability of the low density, highly metastable polymorphs of TTBI. This is clearly an effective method of showing the relationship between known polymorphs and understanding their properties in terms of the energy barriers.

> We thank the reviewer for the very positive feedback. We are pleased that the key motivation and results of the work have come across clearly.

It is a bit surprising that the attempts to show how similar the connected structures are using two similarity measures, atom-centered symmetry functions (ACSFs) and the smooth overlap of atomic positions (SOAP) seems inconclusive. However, this may only reflect the nature of the 1:1 nicotinamide:benzoic acid co-crystal disconnectivity tree structure meaning that the structures are so different. Does the one example justify the third paragraph in the conclusion (p 11 l720) being there rather than in the discussion?

> We examined a second system where the threshold simulations identified two clear superbasins on the energy landscape: tetrolic acid. Similar results were obtained as for the

co-crystal. These results have been added to the supporting information and are now referred to at the end of the section describing the co-crystal results: “Similar results were observed for tetrolic acid (see ESI, Figures S19 - S22): descriptors of local atomic environment do not effectively distinguish between structures belonging to either superbasin identified during the threshold simulations.”

We have also modified the statement in the conclusions: “Although the structures of the energy landscapes for the molecules studied here can be rationalized in terms of intermolecular interactions, we find that for two systems where threshold simulations show clearly separate funnels on the energy landscape (TTBI and tetrolic acid) that the grouping of crystal structures in energy basins is not reproduced by clustering or dimensionality reduction based on commonly-used structural descriptors. Thus, the results are complementary to unsupervised machine learning approaches that have been applied to the analysis of crystal structure landscapes.“

The issue as to whether these methods could automate human judgement of structures whether structures are sufficiently closely related that only the most stable is likely to result from any crystallization process, or the structures may appear as disorder/stacking defects the structure, has to be system-dependent. Interpreting the results of this study also requires insight into the extent of experimental polymorph screening.

We agree. Currently, the methods are best used as an aide to the interpretation of CSP results, or for adding to our understanding of known polymorphs.

The cost of the calculations is not commented upon. It will not be cheap, as at each accepted MC step a multi-stage minimisation is performed to establish whether the structure has gone over the barrier to another structure. This requires the potential energy surface to be described relatively cheaply, but this MC based method is capable of using more accurate atomistic modelling than the recently proposed method of reducing the crystal energy landscape by doing Molecular Dynamics simulations. It is also a requirement that could be speeded up in development of the methodology.

We added a comment on the computational cost on p.3: “With energy minimization performed on all accepted steps (see section 2.3), and similar numbers of accepted steps to the numbers of structures generated during CSP searches, the computational cost involved in performing the threshold simulations is similar to the cost of performing CSP for the molecules studied here.”

The choice of systems is good, within the constraint that the molecules have to be rigid and illustrate the differences between polymorphs with the same or different hydrogen bonding, same or different space-groups. This is pioneering work that is described in sufficient technical detail that the concepts and considerations are clear, so that others could decide whether it would be worth developing this type of approach for their specific system and application.
This paper will influence thinking about how the observation of polymorphs is related to the nature of the potential energy wells, rather than just their depth. It is a challenge to the current practice of

using energy cutoffs in CSP, a practice that does not account for the observation of long-lived highly metastable pharmaceutical polymorphs when solvent removal is very late in the crystallization process (e.g. desolvated solvates). It also shows that there is no quick fix for the problem of reducing the over-prediction problem of lattice energy based organic crystal structure prediction that arises from the neglect of the molecular motion arising from temperature. It clearly shows the conceptual and technological demands of modelling crystals and their transitions at finite temperature. I strongly recommend publication, after the authors have considered the following points:

Please label the horizontal axis in the disconnectivity graphs, explaining the difference in Fig 4, so that it is clear for the other examples.

> We have added the label “number of structures” to the horizontal axis and added the following explanatory text to the captions of figures 4 (a and b), 6, 8, and 11 (a-d) : “The horizontal axis labels the number of structures located during the threshold simulation. The order of structures is chosen to group structures separated by low energy barriers.” In the caption to Figure 4, we have also added “The number of structures differs between (a) and (b) due to the different number of structures sampled in the two space groups.” so that the difference in the number of structures is clearer.

Comparing Table 1 and 2 is difficult on screen. Given that the information can be estimated from Fig 11, consider polishing the TTBI results to be one discussion of the relationship between the polymorphs, and another on the problem of choosing the simulation cell for the algorithm.

> Tables 1 and 2 were designed so that they do not need to be compared directly: the results in Table 1 are summarised in the lower left triangle of Table 2. We prefer to keep the structure of the discussion of TTBI as is. Changing the structure of the discussion would not remove the need to report three sets of transition energies and, so, the comparison of Tables 1 and 2.

The three stage optimization requirement is well explained in S3.1, which reflects the key point, that some transitions can only go via an unphysical mechanism, such as large separation between layers, because of the imposition of translational symmetry. This is a point worth a few sentences in the discussion.

> We have added a sentence in the methods section (section 2.3, page 3) referring to the three-step minimization, and te following to the discussion section, where tetrolic acid results are discussed: “Although there is a clear relationship between the structure of the disconnectivity graph and the strongest interactions between molecules, we cannot necessarily relate the transitions observed during the threshold simulations to physically realistic pathways. In particular, we found that some transitions occur via structures with large separations between layers of molecules (see ESI). These pathways create space for large-scale rearrangement of molecules and might indicate the lack of reasonable single-crystal to single-crystal transition pathways that maintain translational symmetry between some pairs of structures.”

Reviewer #2 (Remarks to the Author):

In this manuscript, the authors present a numerical implementation of the Monte Carlo threshold algorithm for estimating the energy barriers between different polymorphs. They also applied their tool to investigate for different cases with and without space group symmetry constraint. Their results suggests that their tool can adequately sample the energy surface and generate an easily interpretable connectivity map between the local energy minima. An immediate application of this method would be to check if the metastable polymorph can be kinetically stable.

I have only two minor suggestions.
1. It is said the implementation is very similar to basin hopping. Can you give the exact procedure for each MC perturbation. For instance, which variables were perturbed by how many magnitude?. Such details will be useful for the readers to gain a better understanding.

> Additional details have been added to the supporting information (section S1) describing the Monte Carlo perturbations.

2, The authors briefly mentioned their work was inspired from some previous studies on the inorganic systems like MgF_2 and CaF_2. In fact, there have been some similar attempts for estimating the metastability for the newly predicted inorganic compounds (e.g Zhu and Oganov, CrystEngComm 14 (10), 2012). Such work should be mentioned.

> A reference to metadynamics-based approaches has been added in the introduction, including the reference suggested by the reviewer (now reference 23).

Overall, this is a very high quality work in my opinion. It should be published without any doubts.

> Thank you for this positive assessment of the work.

Reviewer #3 (Remarks to the Author):

The work by Shiyue Yang and Graeme Day presents a very interesting approach to identify relevant crystal restructures in crystal energy landscapes by estimating the energy barriers separating the experimental and predicted crystal structures.
From the scientific contribution, the presented approach can be seen as a guideline how to interpret crystal energy landscapes and may be accepted for publication after following points have been addressed:
1. A lot of known information, i.e. the experimental structures, went into the design of the work. In reality, especially for pharmaceutical and other fine-chemical industries, one of the goals of a CSP study is to identify other relevant polymorphs. The authors used the experimental structures and low(est) energy structures as staring points for the threshold simulations. How will the results be affected if other structures are used as staring points? Which structures should be chosen in case the experimental structure(s) has/have not been determined?

We envision different uses of the method. One, which is the focus of the work presented here, is to examine sets of known polymorphs to understand their relationship. We are now exploring the use of the threshold method in the context of predicting structures, where we might not know about all polymorphs of the molecule being studied. In this case, we expect to start simulations from, at minimum, all low energy predicted structures, as well as any further structures that stand out on the energy landscape, such as low energy structures in low density spikes (as for TTBI). The final paragraph of the conclusions has been modified to describe how we see this method being used in different situations:

"We believe that the method presented here is a powerful tool that can be applied in different situations to enhance our understanding of polymorphism. The work presented here has focussed on known polymorphs, where their structures provide the starting points for threshold simulations, which reveal the energetic relationship between the polymorphic structures. More generally, the method could be combined with crystal structure prediction methods without prior knowledge of the observed crystal structures. In this situation, all low energy structures, along with other landmark structures on the predicted energy landscape, could provide staring points and the threshold simulations would produce a global picture of the energy landscape and the molecule's predicted polymorphs."

2. The chosen examples are all based on rigid-body molecules. How will flexibility of the molecules affect this method and the cut-offs that have to be chosen?

The following text has been added on p.3: "Extending the method to molecules with conformational flexibility will require two major modifications: the inclusion of intramolecular degrees of freedom n the Monte Carlo steps and the use of an energy model that treas inter- and intra-molecular interactions during energy minimisation. Cutoffs on intramolecular distortions will need to be determined based on the typical energy changes resulting from different types of intramolecular perturbations."

3. How expensive (computationally) is the additional step compared to the generation of the crystal energy landscapes?

We discuss this in response to reviewer 1.

4. 2.3. Identification of connections between trajectories - concerning the cut-off that was applied to define whether a perturbation that has led to a new energy minimum. How big is the risk that a structure is discharged in case the perturbated unminimized geometry is too far from the minimized geometry/structure? Isn't the applied method at risk of losing structures (basins)?

We should not lose basins if the magnitude of perturbations during Monte Carlo steps is sufficiently small compared to the width of the basins of attraction of individual basins. We chose these to be sufficiently small (as detailed in the supporting information and discussed in the paper) to minimize this risk. The identification of connections between basins relies on robust energy minimisation to locate the nearest (downhill) energy minimum from each perturbed structure. This requirement meant that we spent a lot of time working on the best energy minimisation protocol, which involves three steps (as detailed in the supporting information).

5. 3.1. Sampling within a space group. Even in case two polymorphs adopt the same space group symmetries transformations/connections between those might still be able at a lower energy cost via lower symmetry relations. Was this approach considered in the two chosen examples?

We did not consider this for all examples, but this issue is a large part of the discussion of the tetrolic acid and TTBI sections.

6. 3.2.2. TTBI example: The lattice energy landscape suggests that the most stable polymorph has not been found yet. Is the big energy gap an artifact of the chosen lattice energy minimization method? The lattice energy difference of approx. 50 kJ/mol seems to be high, especially for a fairly rigid molecule and the fact that higher energy structures fall into the same density range.

The global energy minimum structure has been found experimentally and this is being reported in a manuscript that is under consideration elsewhere. We have updated the text to reflect this. The energies of some of the polymorphs are very high for this system and we are finding other molecules where very high energy predicted structures are experimentally realisable (see https://doi.org/10.1039/C9SC02832C and https://doi.org/10.1039/D0TA00219D) We do not believe that the energy gap is an artefact.

7. 3.2.2. TTBI example, last paragraph. The statement “The result is in line with the experimentally observed transformation between the a and b polymorph.” has to be elaborated. How was the value determined (in literature)?

There is not an experimental value for a transition energy. What was meant here is that the results of the threshold calculations agree with the fact that alpha and beta have been observed to interconvert. This is reported in reference 6. We have reworded this sentence to: “The result is in line with the experimental observation that a transformation occurs between the alpha and beta polymorphs”

REVIEWERS' COMMENTS:

Reviewer #1 (Remarks to the Author):

The revision has improved the paper, addressing the concerns in the referee's report. However there a few typos in the new text.

l 204 the inclusion of intramolecular degrees of freedom IN the Monte Carlo steps and the use of an energy model that TREATS inter- and intra-molecular interactions

3.2.1 tetrolic acid should be Tetrolic

l 578 (the four observed polymorphs and the global energy minimum, which we label "), is confusing now the global minimum structure has been found, and should be removed.

Good luck with getting the code released.

Reviewer #3 (Remarks to the Author):

The points raised by the reviewers have been addressed satisfactorily, therefore my recommendations is "publish as is".

Reviewer #1 (Remarks to the Author):

l 204 the inclusion of intramolecular degrees of freedom IN the Monte Carlo steps and the use of an energy model that TREATS inter- and intra-molecular interactions

Done

3.2.1 tetrolic acid should be Tetrolic

Done

l 578 (the four observed polymorphs and the global energy minimum, which we label "), is confusing now the global minimum structure has been found, and should be removed.

Now changed to "Of the five experimentally observed TTBI structures, …
